# Characterization of a recently evolved flavonol-phenylacyltransferase gene provides signatures of natural light selection in Brassicaceae

**DOI:** 10.1038/ncomms12399

**Published:** 2016-08-22

**Authors:** Takayuki Tohge, Regina Wendenburg, Hirofumi Ishihara, Ryo Nakabayashi, Mutsumi Watanabe, Ronan Sulpice, Rainer Hoefgen, Hiromitsu Takayama, Kazuki Saito, Mark Stitt, Alisdair R. Fernie

**Affiliations:** 1 Max-Planck-Institute of Molecular Plant Physiology, Am Mühlenberg 1, 14476 Potsdam-Golm, Germany; 2 Graduate School of Pharmaceutical Sciences, Chiba University, Inohana 1-8-1 Chuo-ku, Chiba 260-8675, Japan; 3 RIKEN Center for Sustainable Resource Science, Suehiro-cho 1-7-22, Yokohama 230-0045, Japan; 4 Center of Plant System Biology and Biotechnology, 4000 Plovdiv, Bulgaria

## Abstract

Incidence of natural light stress renders it important to enhance our understanding of the mechanisms by which plants protect themselves from harmful effects of UV-B irradiation, as this is critical for fitness of land plant species. Here we describe natural variation of a class of phenylacylated-flavonols (saiginols), which accumulate to high levels in floral tissues of *Arabidopsis*. They were identified in a subset of accessions, especially those deriving from latitudes between 16° and 43° North. Investigation of introgression line populations using metabolic and transcript profiling, combined with genomic sequence analysis, allowed the identification of flavonol-phenylacyltransferase 2 (*FPT2*) that is responsible for the production of saiginols and conferring greater UV light tolerance *in planta*. Furthermore, analysis of polymorphism within the *FPT* duplicated region provides an evolutionary framework of the natural history of this locus in the Brassicaceae.

## Abstract (web summary)

Protection from UV-B is critical for land plant survival. Here Tohge *et al.* show that saiginols, a novel class of flavonols that efficiently absorb UV-B, accumulate in *Arabidopsis* accessions collected from high irradiance regions and identify a flavonol phenylacyltransferase gene required for saiginol production.

## Graphical abstract

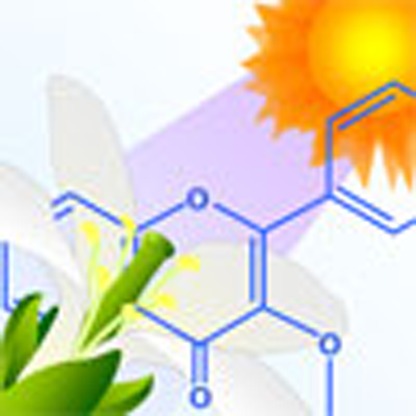

The dependency of plants on sunlight inevitably brings them into exposure to ultraviolet (UV) light, including that in the wavelength range 280–320 nm (UV-B)^1^,^2^. This wavelength range is potentially damaging to DNA, RNA and proteins, and furthermore leads to increased production of free radicals that can activate transposons and cause further mutations^3^,^4^. Thus, although only 0.5% of energy reaching the earth is in this wavelength range^5^, the risk of UV-B damage is profound. For this reason plants have been under considerable natural selective pressure to generate elegant mechanisms to both sense and to respond to the presence UV-B irradiation^1^,^2^,^6^,^7^. The zonal average UV irradiance (flux UV, *F*_UV_) reaching the Earth's surface has continuously increased since 1979 at all latitudes except the equatorial zone^8^ even though the total area of the ozone hole has slightly decreased since 2006, most likely due to efforts restricting usage of chlorofluorocarbons. In addition to its potential impact on natural plant populations, this could greatly impair crop yields since the detrimental effects described above combine to considerably constrain plant seed yields and growth rates. Our understanding of the response to UV-B in land plants has been greatly enhanced by the study of the UV-B resistance 8 (UVR8) UV-B photoreceptor and the downstream cascades under its control^9^. One aspect of the downstream response is the production of additional antioxidant sunscreens including various phenylpropanoids like flavonoids and hydroxycinnamates, and other antioxidants such as ascorbate^1^. The reprogramming of these pathways after exposure to UV-B has been demonstrated to involve independent metabolic responses, and indicates that in *Arabidopsis* seedlings the role of flavonoids is probably of greater quantitative importance than that of hydroxycinnamates, despite the fact that hydroxycinnamates display better absorption of light quanta in the UV-B wavelength range^2^.

Flavonoids and hydoxycinnamates are phenylpropanoids, an important class of plant secondary metabolites that exhibits a range of diverse functions, including roles in protection against biotic and abiotic stresses and developmental processes^10^,^11^,^12^. They are synthesized from phenylalanine via a core pathway that is conserved among land plants, and additional side pathways that start from various intermediates of this core pathway^10^. Whilst some of these, such as the hydroxycinnamate biosynthetic pathways of angiosperms evolved early and are consequently widespread in major plant lineages, others such as the polyphenolic tannins, phenylamines and coumarin derivatives represent relatively recent adaptations and as such are more phylogenetically restricted in their occurrence^10^. Whilst natural variance screening has been carried out in many plant species including *Arabidopsis* with regard to the accumulation of specific primary and secondary metabolites^13^,^14^,^15^,^16^,^17^ relatively few studies have focused on phenylpropanoid metabolism^18^. Given that considerable evidence has accumulated concerning the UV-B protective function of various phenylpropanoids during the processes of flower development, pollination and seed production^19^, we therefore assayed their levels in flowers in a set of 64 *Arabidopsis* ecotypes demonstrated to provide good coverage of the overall natural variability of the species^20^. Here we studied the natural variation of floral secondary metabolites among *Arabidopsis* accessions, characterizing a novel class of phenylacylated-flavonols (saiginols) and a flavonol-phenylacyltransferase 2 (*FPT2*) gene corresponding to altered *in planta* UV-B light tolerance. Furthermore, genomic sequence and gene syntenic analysis of the *FPT* gene duplication region across Brassicaceae species allowed us to develop a picture of the evolutionary framework of the natural history of this locus with respect to natural light selection.

## Results

### *Arabidopsis* accession-specific phenylacylated-flavonols

**Figure 1:**
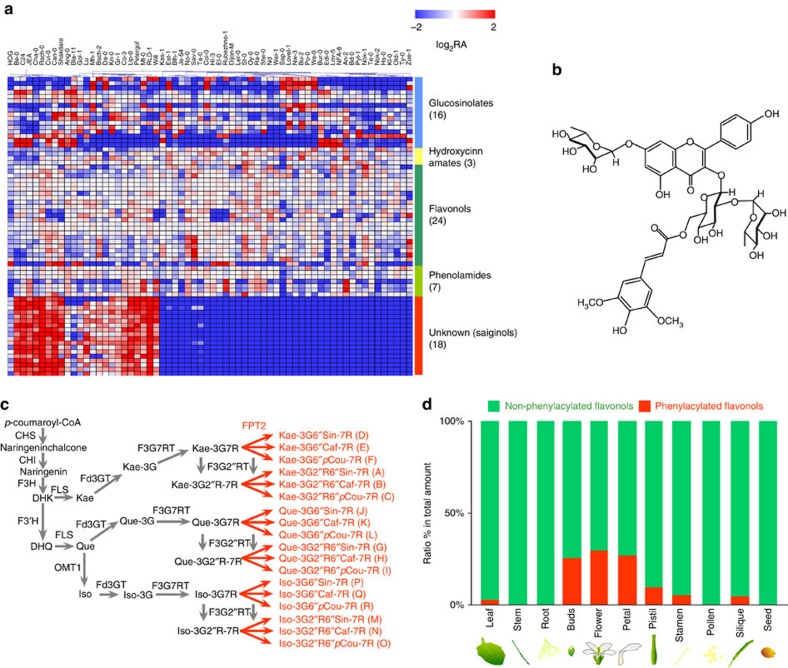
Novel phenylacylated-flavonol glycosides found in *A. thaliana* accessions. (**a**) LC/MS profiling of secondary metabolites in *Arabidopsis* accession flowers. A total of 64 accessions were profiled in biological triplicate (*n*=3). Heatmap shows values displayed on a log_2_ relative peak area (RA) scaled by the average of all values. (**b**) Chemical structure of saiginol A isolated and characterized in this study. (**c**) Constructed novel biosynthetic branches for 18 saiginol productions in producer *Arabidopsis* accessions. (**d**) Tissue specificity of the accumulation of saiginols and relative ratio of phenylacylated(red)/non-phenylacylated(green) flavonols in producer C24 accession. Total phenylacylated- and non-phenylacylated-flavonol contents were compared using their abundance against total amount of all flavonols measured by LC/MS in each tissue type. Caf, caffeoyl; CHI, chalcone isomerase; CHIF3H, flavanone 3-hydroxylase; CHS, chalcone synthase; DHK, dihydrokaempferol; DHQ, dihydroquercetin; F3′H, flavonoid 3′-hydroxylase; Fd3GT, flavonoid 3-*O*-glycosyltransferase; F3G7RT, flavonol-7-*O*-rhamnosyltransferase; F3G2″RT, anthocyanin-3-*O*-glucoside-2″-*O*-rhamnosyltransferase; FLS, flavonol synthase; FPT, flavonol-phenylacyltransferase; G, glucose; Iso, isorhamnetin; Kae, kaempferol; OMT1, *O*-methyltransferase 1; *p*Cou, *p*-coumaroyl; Que, quercetin; R, rhamnose; Sin, sinapoyl.

Application of a previously described liquid chromatography/mass spectrometry (LC/MS) protocol^21^, resulted in secondary metabolite profiles for flowers in a set of 64 *Arabidopsis* accessions and in the detection of a total of 68 peaks consisting of 16 glucosinolates, 3 hydroxycinnamates, 24 flavonoids, 7 putative phenolamides and 18 peaks of unknown chemical structure (Fig. 1a and Supplementary Data 1). Whilst there was considerable difference in the abundance of the peaks corresponding to chemicals of known chemical structure, most striking was the pattern for 18 peaks of unknown chemical structure, which were only present in a subset of the accessions (Supplementary Fig. 1). The abundance of these unknown peaks was highly correlated among unknown peaks (*r*^*2*^=0.6630–0.9997 Pearson's correlation co-efficient, Supplementary Fig. 2), but not with other secondary metabolites such as flavonol glycosides, glucosinolates and hydroxycinnamates with exception of a negative correlation between sinapoyl-glucose (*r*^*2*^=−0.6236 to −0.7117). Among the 64 *Arabidopsis* accessions, 31 accumulated considerable amounts of these compounds whilst 33, including the common laboratory ecotype Col-0, lacked these peaks (Supplementary Fig. 1). MS/MS fragmentation studies suggested that these unknown peaks are novel flavonol derivatives (Supplementary Data 1).

To characterize the chemical structure of these peaks, we re-grew large amounts of the C24 accession (which accumulates these compounds) and purified the corresponding compounds from extractions of ∼30 g FW (fresh weight) of entire flower samples. Following this procedure, we were able to isolate almost 3.5 mg of the major peak, which was then subjected to a suite of analytical chemical procedures, including one- and two-dimensional nuclear magnetic resonance spectroscopy, high-resolution electrospray ionization Orbitrap (ESI-Orbitrap)-MS (in both positive and negative modes) and UV–visible (VIS) spectroscopy (Supplementary Note 1). The combined results of these studies allowed us to identify the compound, which we termed saiginol A, as a novel phenylacylated-flavonol glycoside (flavonol-3-*O*-(2″-*O*-rhamnosyl-6″-*O*-sinapoyl)glucoside-7-*O*-rhamnoside)(Fig. 1b) and to demonstrate that it displayed superior UV-B absorbent properties to phenylpropanoids that contain the flavan ring and one additional phenolic ring structure. On the basis of the core chemical structure and decoration pattern of saiginol A, their retention times and the MS/MS experiments described above we were able to impute the chemical structure (Supplementary Fig. 3) and thus putative biosynthetic paths for the other unknown peaks, which we subsequently collectively refer to as saiginols (Fig. 1c). These peaks, we named as saiginols B-R, are putatively characterized by the presence or absence of three possible aglycones (kaempferol, quercetin and isorhamnetin) and by having one of three types of phenylacylation (sinapoyl, caffeoyl and *p*-coumaroyl moieties) based on annotation by fragmentation of MS/MS and elution time profile (Supplementary Data 1). Evaluation of SciFinder and the *Handbook of Natural Flavonoids* edited by Harborne and Baxter^22^ reveals that the presence of phenylacylated-flavonols is not without precedence with, in addition to the example given above, a total of 29 putative flavonol-phenylacylglycosides being documented in Brassica vegetables^23^. However the Brassica flavonol-phenylacylglycosides found and annotated to date are 2″- or 4″-*O*-phenylacylated-flavonols. Thus, the position to which the phenylacyl and sugar moieties is attached to the saiginols is structurally distinct from all phenylacylated-flavonols reported to date.

Having elucidated the structure of these compounds, we next evaluated their differential accumulation in various tissues. Saiginols were predominantly present in floral tissues but also to a lesser extent in the stem, silique, rosette leaf and the cauline leaf but are essentially, or even totally, absent in senescent leaf and the root (Fig. 1d and Supplementary Fig. 4). Further detailed spatial analysis of flavonol profiles revealed that saiginols predominantly accumulate in the floral petal and to a lesser extent in the stamen and pistil, but are absent in pollen.

### *FPT2* is a key gene for saiginol production

**Figure 2:**
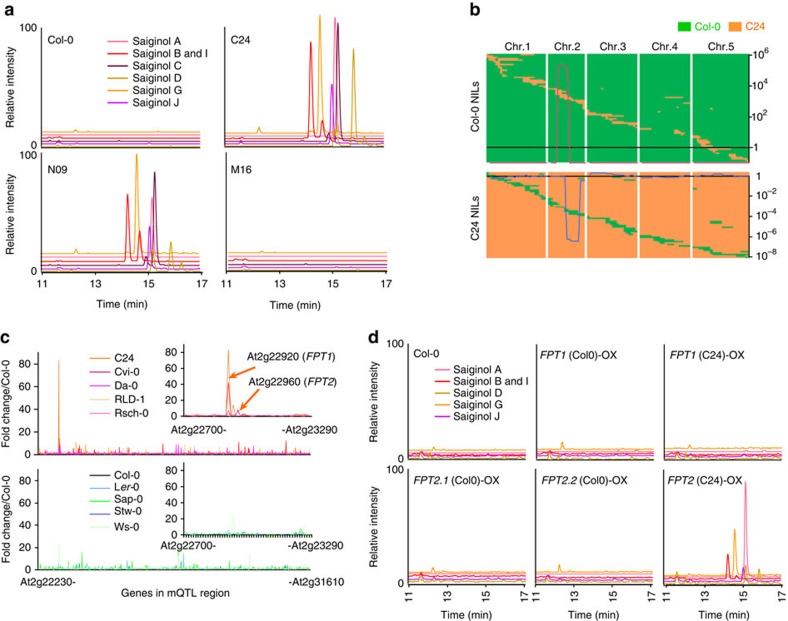
Functional identification of the *FPT* genes based on integrative approach. (**a**) LC/MS chromatogram and (**b**) chromosomal mapping of gain-of-function and loss-of-function NILs obtained from metabolic quantitative trait locus (mQTL) analysis of reciprocal crosses between Col-0 and C24. N and M lines are Col-0 and C24 background introgression lines, respectively. Green and orange boxes indicate Col-0 and C24 DNA segments, respectively. Metabolic QTL was presented by log_2_-transformed relative amount of saiginol A. (**c**) Microarray analysis of gene expression of the genes encoded in the genomic region of chromosome 2 encoding the mQTL in five producing accessions (C24, Cvi, Da, Rsch and RLD) and five non-producing accessions (Col-0, L*er*-0, Ws, Sap and Stw). Intensity indicates fold change estimated by average of expression level in Col-0. (**d**) LC/MS chromatogram showing *in vivo* functional characterization of FPT1 and 2 by metabolite profiling of *35S*-driven overexpressing transgenic plants. All transgenic lines are Col-0 background. *FPT1*(Col-0), *FPT1*(C24), *FPT2.1*(Col-0), *FPT2.2*(Col-0) and *FPT2*(C24) indicate *FPT1* cloned from Col-0 and C24, *FPT2* cloned from Col-0 (*FPT2.1* is shortest transcript and *FPT2.2* is longest transcript) and C24, respectively. Chromatogram showed ion extracted chromatogram for major saiginol A (945 *m/z*), B and I (901 *m/z*), C (885 *m/z*), D (799 *m/z*), G (961 *m/z*) and J (815 *m/z*), respectively.

To identify the genes encoding the protein(s) responsible for the synthesis of these saiginols, we next re-grew previously generated reciprocal introgression lines (near-isogenic line population, NIL) harbouring chromosome segmental substitutions of Col-0 (saiginol non-producer) in C24 (saiginol producer), or substitutions of C24 in Col-0 (ref. 24) and assessed the presence or absence of the saiginols by LC/MS (Fig. 2a). Intriguingly, we found a single loss-of-function line in the C24 population, which showed the overlapping genome region as four gain-of-function lines in the Col-0 population (Fig. 2b and Supplementary Data 2). Given that this genomic region harbours a total of 829 genes (Fig. 2b; At2g22230–At2g31610), we next performed transcript profiling to compare gene expression in the gain-of-function donor line (C24; as well as the producer accessions Cvi-0, Da-0, Rsch-0 and RLD-1) and its recurrent parent (Col-0; as well as the non-producer accessions L*er*-0, Ws-0, Sap-0 and Stw-0; Fig. 2c). Expression levels were generally very similar between the genotypes although a number of genes were significantly different between producing and non-producing accessions including several transposable elements (which comprise 9 of the top 50 most variable genes; Supplementary Table 1). However, when the 829 genes that localized to the chromosomal segment substitution were inspected, only two genes were markedly altered between producing and non-producing accessions. These were annotated in TAIR10 as a serine carboxypeptidase protein like 12 (*SCPL12*, At2g22920) and a pseudo*SCPL* gene (*pSCPL*, At2g22960). We subsequently renamed these genes as putative flavonol-phenylacyltransferases 1 and 2 (*AtFPT1* and *AtFPT2*), respectively. Transcripts for these two genes were more than 17.7 and 6.7 times higher, respectively, in C24 than in Col-0. The fact that they are expressed at exceedingly low levels in Col-0 means that there is unfortunately a paucity of publically available data on their expression. To analyse the flavonol-phenylacyltransferases genes, we cloned the C24 and Col-0 alleles of both genes and evaluated their transcript production. Since the transcript of *FPT2*-Col-0 could not be observed in Col-0 floral material, we cloned *FPT2*-Col-0 from 10-day-old seedlings. Intriguingly, whilst the length of *FPT1* transcript was invariant between Col-0 and C24, the *FPT2*-Col-0 allele encoded four different transcripts *FPT2*-Col-01–4 (743, 657, 637 and 566 bp in length, respectively) and *FPT2*-C24 encoded a considerably longer transcript (1,305 bp).

With the purpose of testing the function of various FPT proteins experimentally, we performed complementation assays in Col-0 and knockout *fpt1* and *fpt2* mutants. CaMV 35S driven complementation with the C24 allele of *FPT2* resulted in the production of all 18 saiginols in flowers of the transgenics. However, neither expression of the Col-0 allele of *FPT2* in the Col-0 *fpt2* knockout line nor expression of the Col-0 or the C24 allele of *FPT1* in the Col-0 *fpt1* mutants yielded flowers, which produce saiginols (Fig. 2d and Supplementary Fig. 5). Complementation experiments also revealed that FPT2 expression corresponds to the production of the flavonol-phenylacylglucosides, but FPT1 expression did not. The saiginol levels in *FPT2*-C24-overexpressing and Col-0/C24 NIL lines revealed differences in floral tissues (The NIL lines showed 10 times higher accumulation than the *35S*-driven transgenic plants).

### Saiginols accumulation correlates with UV light tolerance

**Figure 3:**
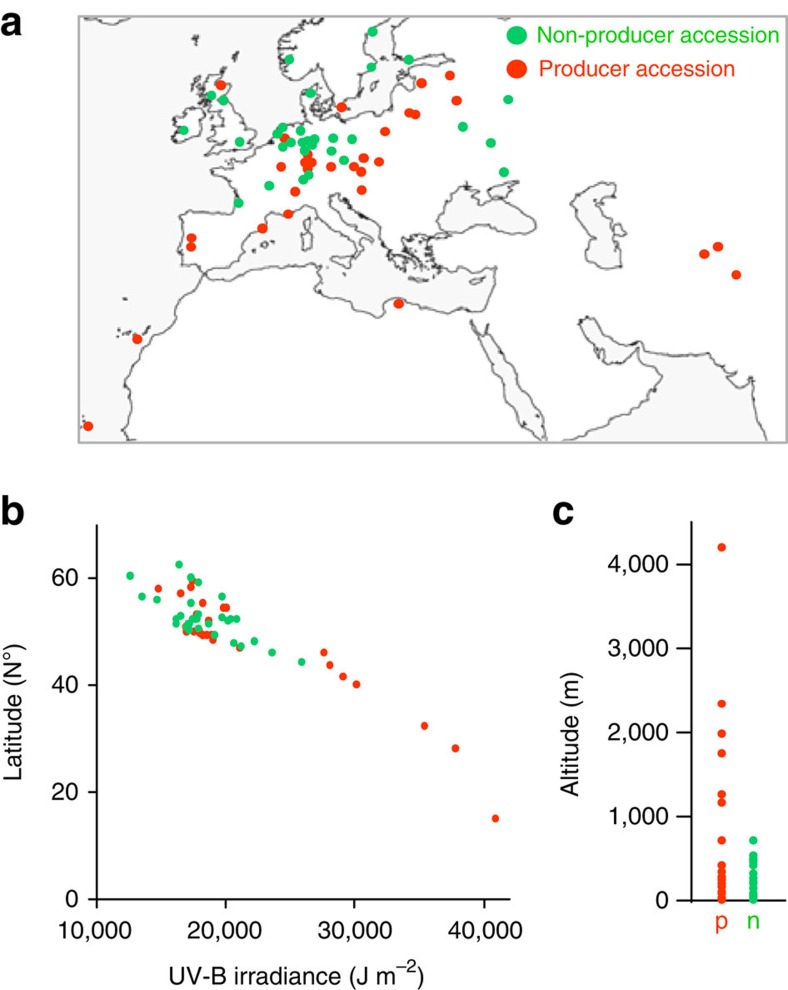
Geographical distribution of *A. thaliana* accessions used in this study. (**a**) Geographical distribution of producers (orange) and non-producers (green) in the world scale map. A total of 63 *A. thaliana* ecotypes are mapped. (**b**) Plots of producers and non-producers (by latitude (N°) and UV-B irradiation (J m^−2^) averaged of daily UV-B irradiance during 1985 and 2005 estimated from satellite data (Soda (http://www.soda-is.com/eng/index.html)). *r*^2^=0.8624 (producers) and 0.4476 (non-producers). (**c**) Plots of producers and non-producers by altitude (m) estimated by Shuttle Radar Topography Mission (http://www2.jpl.nasa.gov/srtm/). Map by FreeVectorMaps.com. Green and orange indicate non-producing and producing accessions, respectively.

To place the occurrence of these novel compounds in an eco-physiological context, the producers and non-producers were mapped on a world map according to their site of origin (Supplementary Table 2). Intriguingly, although the sample number is admittedly relatively small, all of the accessions deriving from high irradiance habitats produce these metabolites. By contrast, in low irradiance areas such as mid and north Europe habits and lower altitude (Fig. 3a), both producing accessions and non-producing accessions can be found. Thus, the presence of the phenylacylated-flavonols may well be selected for in populations exposed to higher UV-B irradiance only in high light irradiance regions. The average of daily UV-B irradiance during 1985 and 2005 was assessed for the region of origin of the accession from satellite data (obtained from Soda (http://www.soda-is.com/eng/index.html). These data alongside latitude data were plotted in Fig. 3b which shows that there is clear relationship between at UV-B irradiance above 26,000 J m^−2^ where only producers are found. In addition, plots of the altitude calculated by Shuttle Radar Topography Mission (http://www2.jpl.nasa.gov/srtm/) showed that the accessions isolated from above 1,000 m altitude are exclusively saiginol producers (Fig. 3c and Supplementary Fig. 6).

**Figure 4:**
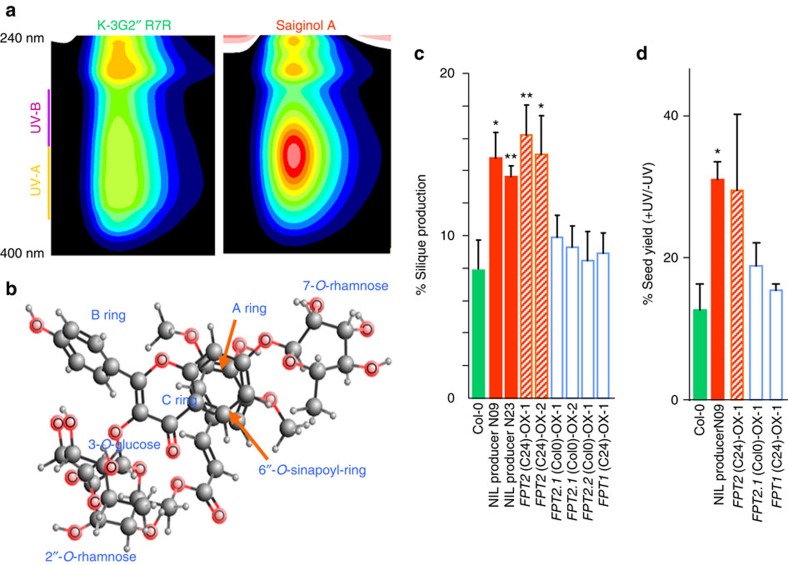
Functional characterization of the role of saiginols and *FPT2* in protection against UV-B irradiation. (**a**) UV absorbance shift of flavonol by phenylacylation measured by HPLC-PDA. Purified compounds, a precursor (K-3G2″R7R) and saiginol A were used for the profile. (**b**) Computational estimation of the most stable stereochemical structure performed by MMFF94 of Marvin (http://www.chemaxon.com/). (**c**) Rate of silique production under UV-B irradiation after long-term (2 h per day) UV-B treatment (1 W m^−2^) using the detached immature inflorescences of first bolting. Error bars indicate the s.e. of six biological replicates. (**d**) Seed yield after long-term (2 h per day for 28 days, 2 W m^−2^) UV-B treatment using 3-week-old intact plants. Seeds were collected on a single plant basis but three individual plants per genotype were measured. Data are presented as mean±s.e.m. *P<0.05, **P<0.05.

The UV–VIS spectra of saiginol A (kaempferol-3-*O*-(2″-*O*-Rha-6″-*O*-sinapoyl)Glc-7-*O*-Rha), revealed *λ*max at 206, 223, 267 and 332 nm (Supplementary Note 1), indicating that the phenylacylated-flavonol is much more efficient in UV-A/B absorption than its precursor, kaempferol-3-*O*-Glc-2″-*O*-Rha-7-*O*-Rha (Fig. 4a) (*λ*max at 202, 249 and 266 nm)^25^, which is most abundant flavonol in *Arabidopsis thaliana*. Such an absorption addition by one more phenolic ring (for example, from sinapoyl-donor, sinapoyl-Glc, *λ*max at 203, 241 and 329 nm)^25^ in flavonol decoration has previously been reported for 3″,6″-di-*O*-(*p*-coumaroyl)isoquercitrin in Scots pine^26^ which is a similar, although structurally distinct, molecule to those described here. This thus presents the possibility that the synthesis of saiginols in Scots pine and *Arabidopsis* may have occurred as a result of convergent evolution. Furthermore, computational estimation of the most stable stereochemical structure of saiginol A using MMFF94 resulted in a bridge piled structure between the aromatic rings A and sinapoyl-ring (Fig. 4b) similar to that of the intramolecular co-pigmentation of phenylacylated-anthocyanins reported in Scots pine^27^. Previous research of absorption shift by intermolecular co-pigmentation of *p*-coumaroyl-isoquercitrins in Scots pine suggested that phenylacylation into 6″-*O*- position indicates a further enhancement of its absorption at UV-B range. Importantly, neither of these compounds absorb in the photosynthetically active radiation range meaning that their presence does not result in a trade-off against photosynthetic efficiency.

To assess the role of FPT2 and by implication the role of saiginols, in conferring additional protection against UV-B, we grew the overexpressing lines for both alleles of *FPT1* and *FPT2* alongside Col-0 the gain-of-function NIL and subjected them to a range of UV-B treatments. A difference between these genotypes most likely directly reflects the role of saiginols as we do not see major changes in the accumulation of other flavonols in either natural accessions or overexpressors. Nevertheless we cannot formally rule out that differences in FPT2 expression may be correlated with other phenotypic effects that influence UV-B sensitivity independently of saiginols. Since saiginols accumulate in floral tissue especially in buds and petal but not in pollen, although pollen is extremely sensitive to UV-B irradiation, we hypothesize that saiginols may protect pollen or its haploid state from UV-B light before flower opening and pollination. We, therefore, ensured using a detached flower experiment^28^ that the flower organs were equidistant from the UV-B source. As documented in Fig. 4c and Supplementary Fig. 7, both NIL producers (N09 and N23) and *FPT2*-C24-overexpressing lines showed significantly higher silique production following UV-B treatment, whilst no significant higher silique production in normal condition in saiginol-producing lines. We next assessed the total seed yield of intact plants at maturity. Although, as is typical of such experiments, there was a high variability between replicates, the experiments revealed a consistently significantly higher seed yield of the gain-of-function NIL. In addition, *FPT2*-C24-overexpressing line that exhibit relatively lower accumulation of saiginols in floral tissues (Supplementary Fig. 5) was characterized as exhibiting higher seed yield to the control plants under UV-B irradiation. Consistent with their lack of saiginols, lines overexpressing the *FPT1* alleles and the *FPT2*-Col-0 allele displayed similar seed yield following UV-B treatment to those in control lines (Fig. 4d).

### Deletion of *FPT2* gene in *SCPL* duplication region

**Figure 5:**
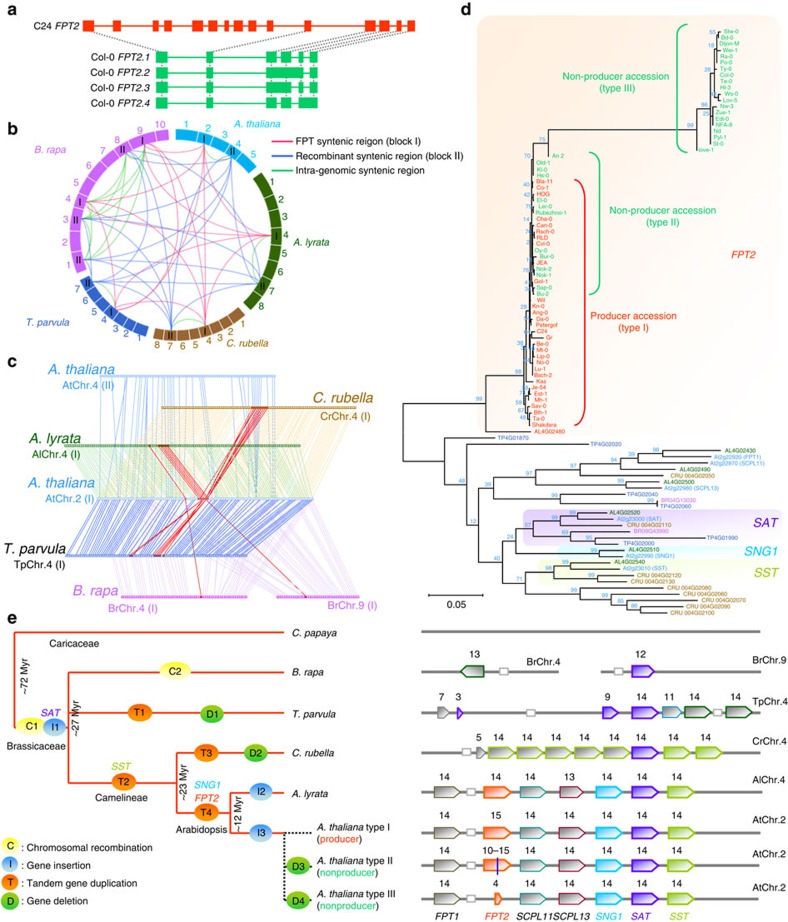
Genomic structure and sequence analysis of *FPT* gene cluster region in plant species. (**a**) Gene deletion identified in the *FPT2*-Col-0 genomic sequence. Coding sequence including splicing variance found in Col-0 and C24 was mapped with their genomic sequence. (**b**) *FPT* gene syntenic regions found in plant species. I, red, syntenic block I (containing *FPT* gene cluster); II, blue, syntenic block II (recombinant syntenic region); green, linkage of intra-genomic syntenic region. (**c**) Syntenic gene linkage between plant species. Red box and line indicate *FPT*/*SCPL* genes. Similarity between *AtSCPL* and *SCPL* genes obtained from Brassicaceae species is evaluated by TAIR-BLAST (https://www.arabidopsis.org/). (**d**) Phylogenetic analysis using CDS of *SCPL* genes in syntenic region of Brassicaceae species. A short gene *TP4G01860* was excluded from this analysis. The phylogenetic tree was created using MEGA5.2 (ref. 60). Scale implies nucleotide substitutions per site. (**e**) Hypothetical scheme of evolutional events in *FPT* gene cluster region among Brassicaceae species. The taxonomical tree of Brassicaceae species was constructed via reference to PLAZA (http://bioinformatics.psb.ugent.be/plaza/) based on NCBI taxonomic analysis (http://www.ncbi.nlm.nih.gov/taxonomy). *FPT* tandem duplication cluster of *SCPL* genes including *FPT1* and *FPT2*. Numbers above genes indicate number of exons. Red, yellow and blue indicate sub-clades found by phylogenetic trees analysis in **d**. C1 and C2, chromosomal recombination in Brassicaceae and *B. rapa*, respectively; I1, hypothetical time of *SAT* insertion into *FPT* region; I2, gene insertion into fourth and eleventh exons of AL4G02540; I3, gene insertion into second intron of *FPT2*; T1–4, tandem gene duplication in *T. parvula*, Camelineae (*C. rubella, A. lyrata* and *A. thaliana*), *C. rubella* and *Arabidopsis* (*A. lyrata* and *A. thaliana*), respectively; D1–4, gene deletion in *T. parvula*, *C. rubella*, *A. thaliana* non-producer type II and type III.

Having established that full-length *FPT2* from C24 functions to enhance UV-B protection, we next sought to clarify the molecular reason for the natural variance for the presence of the saiginols. We first performed genomic sequencing of *FPT1*-C24 and *FPT2*-C24 and mapped their full-length cDNA sequences obtained from Col-0 and C24. Analyses of *FPT1* revealed a deletion (∼600 bp) in the *FPT1*-C24 promoter region and an insertion (∼10 and 6 bp) within the first and second introns of *FPT1*-C24 (Supplementary Fig. 8). The protein-coding sequences, however, were highly similar and comprised genome sequence with complete match of all 14 exons. On the other hand, genomic sequence analyses of *FPT2* (Col-0, 2,039 bp; C24, 4,262 bp) revealed a large gene deletion (∼2,279 bp) in Col-0, spanning the region corresponding to the second to eleventh exons of *FPT2*-C24 (Supplementary Fig. 9). This analysis indicated that the four transcripts of *FPT2*-Col-0 described above are alternative splicing variants (Fig. 5a). One of the splice variants (At2g22960) was previously identified as a pseudo*SCPL* gene since the ATPase-like sequences upstream of the first exon suggest that this *SCPL* gene lacks a promoter^29^, however, importantly the predicted translation products of all four splice variants terminate shortly after the putative catalytic Ser (ref. 30), and thus lack the active site Asp and His residues. Thus, it is highly likely that FPT2-Col-0 does not a function as a phenylacyltransferase *in vivo*. Furthermore, the result of BLAST search of this deleted sequence (2,279 bp) revealed that the deleted sequence has similarity to *SCPL10* (At2g23000, *SAT*, anthocyanin sinapoyltransferase) in the coding sequence, as well as one fragment of copia-like retrotransposon in its untranslated regions. The *FPT* genomic region in chromosome II (AtChr.2) is very close to the *SCPL* cluster region generally discussed as containing five *SCPL* genes (*SCPL11*, At2g22970; *SCPL13*, At2g22980; *SCPL08*, *SNG1*, At2g22990; *SCPL10*, *SAT*, At2g23000; and *SCPL*09, *SST*, At2g23010) of which three to date have been assigned specific enzymatic activities, namely sinapoyl-glucose: malate sinapoyltransferase 1 (SNG1); sinapoyl-glucose: anthocyanin sinapoyltransferase (SAT); and sinapoyl-glucose: sinapoyl-glucose sinapoyltransferase (SST)^30^. Interestingly, all *SCPL* genes have 14 exons except *FPT2*-C24, which has 15 exons due to a short insertion in second exon, but *FPT2*-Col-0 has just 4–6 exons (Supplementary Fig. 10). This result revealed that the difference between *FPT2*-Col-0 and *FPT2*-C24 is the result of transpositional gene deletion as opposed to an insertional gain of function. The shared number of exons in this *FPT* duplication cluster, as well as the similarity of the gene sequence between *FPT* genes and *SCPL* later in this cluster, suggest that this tandem gene duplication in *FPT* region occurred before the transpositional gene deletion of *FPT2* in non-producing accessions.

We next extended the coding region sequencing across further all 64 accessions and were able to identify a strict relationship between this polymorphism and saiginol production among the accessions. For this purpose sequences were obtained using two primer sets one designed for the full-length coding region and the other to include 71 bp forward upstream and 55 bp reverse downstream of deleted region for a total of 44 ecotypes. Further, sequences were evaluated and constructed with publically available sequence (*Arabidopsis* 1,001 genome (http://signal.salk.edu/atg1001/3.0/gebrowser.php). A total of three classes of genotype were observed: type I, producer, similar to C24 (31 accessions); type II, non-producer, similar to C24 but with a 2 bp deletion in the eleventh exon (L*er*-0, Sap-0, Bur-0 and Rubezhno-1) with 14 bp deletion in third exon (Nok-1 and Nok-2), deletion of seventh exon (El-0, Oy-0 and Bu-2) and deletion in the seventh to tenth exons (Hs-0, Kl-0, Old-1 and An-2); and type III, non-producer, similar to Col-0 (deletion in the second to tenth exons, 18 accessions) and similar to Col-0 but with a 9 or 72 bp deletion in the tenth exon (Stw-0 and Bd-0) (Supplementary Fig. 11). The inactivation pattern does not reflect geographic demography of accessions, for example, accessions L*er*-0, Sap-0, Bur-0 and Rubezhno-1, which have 2 bp deletion in the eleventh exon from Poland, Czech Republic, Ireland and Ukraine, respectively. The fact that a large or short deletion in the coding region of *FPT2*, was always and exclusively observed in saiginol non-producing genotypes (Supplementary Fig. 11), suggests that this deletion invokes the observed natural diversity of saiginol production. That said the presence of such a large variety of genomic polymorphism across *Arabidopsis* accessions suggests that the gene deletion within the *FPT*–*SCPL* region is a relatively recent event.

### Genomic signatures and evolutionary framework in *FPT* region

To assess the evolutionary context of the *FPT2* gene deletion within the *SCPL* cluster region, we first performed cross-species genome-wide analysis to identify gene conservation and syntenic regions of *FPT2*. Results of a PLAZA (http://bioinformatics.psb.ugent.be/plaza/) search and further genomic sequence analysis suggest that the sequence of *FPT2* is not found in other plant species with the exception of *Arabidopsis lyrata* AL4G02480, although several orthologues of other *SCPL* genes have been found in several plants especially those of the Brassicaceae. We next carried out a syntenic cross species orthologue analysis of the *AtFTP* region. Syntenic blocks (defined as block I) were found in AlChr.4 (seven *SCPL* genes in *A. lyrata*), CrChr.4 (nine *SCPL* genes in *Capsella rubella*), TpChr.4 (seven *SCPL* genes in *Thellungiella parvula*), BrChr.4 (one *SCPL* gene in *Brassica rapa*) and BrChr.9 (one *SCPL* gene in *B. rapa*)(Fig. 5b, Supplementary Fig. 12 and Supplementary Data 3 and 4). Furthermore, intrasyntenic regions (defined as block II regions see Supplementary Figs 12 and 13, and Supplementary Data 4) possibly caused by chromosomal recombination were found in AtChr.4, AlChr.7, CrChr.7, TpChr.7, BrChr.1, BrChr.3 and BrChr.8 (Fig. 5b and Supplementary Fig. 12), but do not contain *SCPL* genes. However, papaya does not have such a syntenic region in its genome but rather has two short similar blocks, which share genes with both AtChr.2 and AtChr.4, but do not harbour *FPT*. The identified syntenic blocks in other Brassicaceae species, however, fit to previously reported segmental collinearity between *A. thaliana* and those species^31^,^32^,^33^,^34^,^35^. This observation suggests that *FPT* genes, like all members of the *SCPL* family, evolved after chromosomal recombination split this block across two chromosomes. Further detailed syntenic block analysis revealed that numbers of total *SCPL* genes in this syntenic region are different between species except *A. thaliana* and *A. lyrata* (Fig. 5c and Supplementary Fig. 14). This suggests that *SCPL* genes were not duplicated in same manner and that *SCPL* gene duplication occurred after speciation. To test metabolic conservation of FPT2 in *Arabidopsis* species including *A. thaliana* and *A. lyrata*, we performed LC/MS profiling of saiginols in *A. lyrata* and *C. rubella*. Saiginol A was clearly present in *A. lyrata* but not in *C. rubella* suggesting that FPT2 evolved after speciation between *Arabidopsis* and *C. rubella* (Supplementary Fig. 15). Phylogenetic analysis of the *SCPL* genes of the block I syntenic region in Brassicaceae species and *FPT2* in all *A. thaliana* accessions studied here revealed that anthocyanin-sinapoyltransferase (*SAT*) appears to be the first *FPT* inserted into this region because only this gene is well conserved across the Brassicaceae (Fig. 5d). By contrast, *FPT1* and *SCPL11* genes appear to have evolved relatively recently since only *A. lyrata* harbours an orthologue of these genes. Further phylogenetic analysis using predicted protein sequences also supports similarity between them (Supplementary Figs 16 and 17). In addition it appears that the *C. rubella SCPL* was duplicated from sinapoyl-glucose: *SST* gene subsequent to its speciation from *Arabidopsis*.

## Discussion

Our hypothesized evolutionary framework for genes in this gene cluster is summarized in Fig. 5e with speciation tree based on NCBI taxonomic analysis. The presence/absence of *SCPL* within the intrasyntenic region demonstrates that the origin of *SCPL* likely occurred after speciation of the Caricaceae and Brassicaceae within the Brassicales that is, around 72 million years ago (Myr ago)^36^. Between 27 and 72 Myr ago^37^, inter-chromosomal recombination (between C1 as described in Fig. 5e) occurred independently within Brassicaceae species. Subsequent to chromosomal recombination, the first *SCPL* appeared in syntenic block I (I1) ultimately evolving into *SAT* (anthocyanin sinapoyltransferase) gene. Furthermore, 23–27 Myr ago after speciation to Camelineae/Capsella^37^, in *B. rapa* a second chromosomal recombination occurred (C2), whereas *FPT* was tandemly duplicated in *T. parvula* (T1). On the other hand, *SST* gene was created by tandem gene duplicated in this cluster (T2). During the relative recent period 12–23 Myr ago, *FPT1* and *FPT2* genes occurred by tandem gene duplication (T4) only on the origin of the *Arabidopsis* species, since tandem gene duplication in *C. rubella* occurred in different manner. The observed variation of genotypes among non-producing *A. thaliana* accessions suggests that *FPT2* gene has been deleted or differentially mutated. However, it is important to note that although the mechanisms resulting in the non-production of saiginols differ the phenotypes, they confer that is, robustness in the face of high UV irradiance is the same most likely indicating exposure to similar selective pressure. Interestingly, classification of *A. thaliana* accessions based on function/non-function of *FPT2* genes was not similar to any other classification of accessions based on criteria such as global single-nucleotide polymorphisms, cold response, salt tolerance, pathogen response or glucosinolate content^38^,^39^,^40^,^41^,^42^. This observation suggested that the evolutionary events have occurred individually and have been differentially filtered by natural selection. This fact suggests that the pressure of natural light selection was independent of that occurring for the other environmental natural selection factors listed above.

We demonstrate here that the FPT2 protein corresponds to one step of a multi-step pathway for the production of the 18 newly identified and annotated saiginols, which have enhanced UV-B-absorbing properties to non-phenylacylated-flavonol-glycosides. As such this is another example, similar to that of the recently described alpha-pyrones^43^, of a rapidly evolved biochemical pathway, which exploits the pre-existing metabolic infrastructure. In this instance, 18 novel phenylacylated-flavonols are produced following neofunctionalization of the *FPT2* gene after gene duplication, possibly through divergence of substrate specificity. Phenylacyltransferases are not uncommon in plant natural product biosynthesis. Members of the BEAT-AHCT-HCBT-DAT (BAHD) family are collectively responsible for the acylation of a wide range of compounds such as flavonoids, hydroxycinnamates, terpenoids and alkaloids being very well characterized. Three BAHD anthocyanin phenylacyltransferases from Gentiana^44^, *Arabidopsis*^45^ and Tomato^46^ have been characterized, but no flavonol-phenylacyltransferase have been reported previously. The serine carboxypeptidase-like (SCPL) acyltransferase family was more recently identified and characterized in both *Arabidopsis* and oat, and is involved in the acylation of anthocyanins, sinapoyl-derivatives, glucosinolates and the important defence compound and triterpene saponin, avenacin^47^,^48^. While BAHD enzymes use CoA-thioesteters as the acyl donor, SPCL enzymes use *O*-glucose esters^48^. To date, four *SCPL* genes have been characterized as encoding specific sinapoyltransferases of malate (SNG1), choline (SNG2), sinapoyl-glucose (SST) and anthocyanin (SAT) in *Arabidopsis*. Intriguingly, FPT2 is proposed to catalyse a highly similar (in terms of position and acyl-donor) phenylacylation, to that carried out by the *Arabidopsis* anthocyanin phenylacyltransferase, A3G6″*p*CouT, of the BAHD family^45^,^49^. However, the precise biological function of the phenylacylated-anthocyanins are as yet unknown.

Characterization of FPT2 in this study suggests it is capable of catalysing several single steps of a multi-step reaction with substrate flexibility for both acceptors and donors of the phenylacyl moiety. Such an enzyme potentially involved in such single steps of a multi-step reaction supports the suggestion that a competitive advantage is conferred to genotypes, which produce saiginols and that flavonol decoration may represent a powerful tool for enhancing seed yield in crops. In addition, analysis of the origin of producing and non-producing accessions revealed that the presence of a functional FPT2 seemingly confers a selective advantage in high light growth habits. Furthermore, analysis of polymorphism within the *FPT* duplicated genomic region provides an evolutionary framework of the natural history and current status of this locus in the Brassicaceae.

## Methods

### Plant materials

*A. thaliana* accessions used in this article were described in Supplementary Table 2. Plants were cultured on agar plate in a growth chamber under standard long day light conditions (16 h day, 140–160 μmol m^−2^ s^−1^, 20 °C; 8 h night, 16 °C) for 14 days and transferred to soil (type GS-90 Einheitserde; Gebrueder Patzer). Materials were collected from individual plants, immediately frozen in liquid nitrogen, and stored at −80 °C until further use. *A. lyrata* (MN47) and *Capella rubella* (MTE) used in this article were grown in a greenhouse under control conditions, and were immediately frozen with liquid nitrogen after harvest.

### Secondary metabolite profiling using LC/MS

Metabolite profiling of secondary metabolites were performed by the method described by Tohge *et al.*^21^. Ground frozen flowers were aliquoted and homogenized in 20 μl of extraction buffer (80% MeOH, 5 μg ml^−1^ isovitexin as an internal standard) per milligram of fresh weight of tissue in a mixer mill for 3 min at 25 Hz with zirconia ball. After centrifugation at 12,000*g*, the supernatants were immediately used for secondary metabolite profiling. Secondary metabolite analysis was performed on HPLC system Surveyor (Thermo Finnigan, USA) coupled to Finnigan LTQ-XP system (Thermo Finnigan, USA). All data were processed using Xcalibur 2.1 software (Thermo Fisher Scientific, Waltham, USA). Peak identification and annotation were performed with a combination approach using standard chemical confirmation^25^, MS/MS profiling, retention time profiling, mutant analysis^50^,^51^ and literature survey^21^,^49^,^50^,^52^,^53^,^54^,^55^. To carry out mutant analysis for flavonoid derivatives, 14 mutants; *ugt78d2* mutant (flavonoid-3-*O*-glucoside-less)^49^, *tt7* mutant (quercetin and isorhamnetin derivative-less)^50^, *ugt78d1* mutant (flavonol-3-*O*-rhamnoside-less)^52^, *ugt78d3* mutant (flavonol-3-*O*-arabinoside-less)^56^, *omt1* mutant (isorhamnetin-derivative-less)^50^, *ugt89c1* mutant (flavonol-7-*O*-rhamnoside-less)^57^, *tt4* mutant (all flavonoid-less)^58^ and *pap1*-D mutant (anthocyanin-overaccumulator)^49^,^59^, and La-*er* background *tt* mutant series obtained from NASC (*tt3*,N84; *tt4*, N85; *tt5*, N86; and *tt6*, N87) were used.

### Procedure of purification and characterization of saiginol A

Column chromatography was carried out over ODS (Nacalai Tesque, Cosmosil 75C_18_-OPN). HPLC analysis was carried out on an Atlantis (*φ* 4.6 × 150 mm, Waters) at a flow rate of 0.5 ml min^−1^. Preparative HPLC was performed on a LC 10A system (Shimadzu) using an Inertsil ODS-EP 5 μm (*φ* 6.0 × 150 mm) at 30 °C and monitoring was accomplished by photodiode array detector (PDA) (200–600 nm). HPLCPDA/ESIMS was performed on a Finnigan LCQ-DECA mass spectrometer (ThermoQuest, San Jose, CA, USA) and an Agilent HPLC 1100 series (Agilent Technologies, Palo Alto, CA, USA) (Tohge *et al.*^49^). HR-ESI-MS was performed on an Exactive mass spectrometer (ThermoQuest, San Jose, CA, USA). Optical rotations were determined on a JASCO P-1020. UV spectra were recorded on a JASCO V-560. Nuclear magnetic resonance data were recorded on JEOL JNM ECP-600. The deuterated solvent CD_3_OD was used for peak 4. Coupling constants are expressed in Hz.

Plant samples (FW, 26.72 g) were collected and immediately frozen in liquid nitrogen, the whole of which was immediately extracted with methanol. After concentration, MeOH liquid extraction was extracted with *n*-hexane, CHCl_3_ to remove low-polarity metabolites. After the liquid–liquid partition and concentration, MeOH soluble fraction was obtained and was dissolved with H_2_O. After liquid–liquid partition with *n*-BuOH, *n*-BuOH fraction was obtained (256.9 mg). This fraction was applied to ODS column (*φ* 3.5 × 7 cm), and roughly separated by eluting with a gradient of H_2_O as solvent A and CH_3_CN as solvent B and the following elution profile (fraction 1: 0% CH_3_CN; fraction 2: 10% CH_3_CN; fraction3: 20% CH_3_CN; fraction 4: 30% CH_3_CN; fraction 5: 40% CH_3_CN; fraction 6: 50% CH_3_CN; fraction 7: 60% CH_3_CN; fraction 8: 70% CH_3_CN; fraction 9 80% CH_3_CN; and fraction 10: 100% CH_3_CN (elution solvent: 70 ml per fraction)) to give 10 fractions. After LC/MS analysis for trace of peak 4, fraction 2–4 was assembled (fraction A). Fraction A was applied to ODS column (*φ* 3.5 × 7 cm) again, and separated by eluting with a gradient of H_2_O as solvent A and CH_3_CN as solvent B and the following elution profile (fractionA-1 and A-2: 0% CH_3_CN; fraction A-3 and A-4: 10% CH_3_CN; fraction A-5 to A-7: 20% CH_3_CN; fraction A-8 to A-11: 30% CH_3_CN; and fraction A-12: 100% CH_3_CN (elution solvent: 30 ml (fraction A-1 to A-5) and 15 ml (fraction A-6 to A-12))) to give 12 fractions. After LC/MS analysis for trace of peak 4, fraction A-10 and A-11 were assembled (fraction B). Fraction B (12.0 mg) was applied to preparative HPLC using an isocratic elution (20% CH_3_CN in H_2_O) at a flow rate of 4 ml min^−1^ to give peak 4 (3.5 mg).

### Profiling UV–VIS spectrum of saiginol A and K-3G2″ ***R7R***

Saiginol A and *K-3G*2″*R7R* (kaempferol-3-*O*-(-2″-*O*-rhamnosyl)glucoside-7-*O*-rhamoside, which were purified in this study and previous work^25^, were subjected to HPLC-PDA analysis. HPLC analysis was performed on a Summit HPLC system (Dionex, Idstein, Germany) using an Luna C18_(2)_ (*φ* 2.0 × 150 mm, Waters) at 25 °C and monitoring was accomplished by PDA-100 photodiode array detector (Dionex, Idstein, Germany) (190–600 nm). Peaks were separated by eluting with a gradient described previously^21^.

### Metabolite QTL analysis for saiginols

Introgression line of NILs was obtained as described previously^24^. Inflorescences from 5-week-old *Arabidopsis* plants were collected from three individual plants of 45 M lines (C24 background) and 69 N lines (Col-0 background) for metabolite profiling of secondary metabolites.

### Microarray analysis

Transcriptome analysis was carried out using ATH1 microarrays as described previously^49^ with five producing accessions (C24, Cvi, Da, Rsch and RLD) and five non-producing accessions (Col-0, L*er*-0, Ws, Sap and Stw). Duplicate hybridizations were carried out for Col-0 and C24, and a single hybridization was performed for all other accessions. Intensity indicates fold change estimated by average of expression level in Col-0.

### Overexpression of *FPT1* and *FPT2* genes

*FPT1* and *FPT2* overexpression constructs were created by cloning the full-length cDNAs of *FPT1* and *FPT2* genes from Col-0 and C24 *Arabidopsis* under the control of the CaMV *35S* promoter in vector pK7GW2 (Invitrogen), a binary vector with a gateway cassette, using the In-fusion HD cloning kit (Takara). Binary plasmids were transferred to *Agrobacterium tumefaciens* GV3101 (pMP90) and transformed to *Arabidopsis* plants (Col-0) and T-DNA insertion lines (SALK_067799 and SALK_111019) according to the floral dip method. Transgenic plants were selected with 50 mg l^−1^ kanamycin sulfate for pK7GW2 and T4 progenies were used for the analysis. Primers used for the cloning are described in Supplementary Table 3. Full-length cDNA sequences of *FPT1*(Col0), *FPT1*(C24), *FPT2*(Col0) and *FPT2*(C24) are shown in Supplementary Figs 8, 9 and 11.

### Full-length cDNA and genomic sequence of *FPT2* genes

Full-length cDNA of *FPT2* from 64 *A. thaliana* accessions and genomic sequences of *FPT2* from 2 *Arabidopsis* accessions (Col-0 and C24) were sequenced. Primers used for amplification and sequencing of *FPT2* are described in Supplementary Table 3. All primers were designed using NCBI blast primer (http://www.ncbi.nlm.nih.gov/tools/primer-blast/).

### Silique and seed productions under UV-B irradiation

For silique production experiment, inflorescences from the primary bolts of 5-week-old *Arabidopsis* plants were used. The detached inflorescences were irradiated with UV-B light (1 W m^−2^) for 2 h per day during midday time (5–7 h after the onset of normal light) for 14 days by placing their cut ends in wells of a 96-well microtitre plate containing water. For seed yield experiment, 3-week-old *Arabidopsis* plants were irradiated with UV-B light (1 W m^−2^) for 2 h per day during midday time (5–7 h after the onset of normal light) for 28 days.

### Synteny analysis and gene duplication of *FPT* genes

To compare the genomic context of *Arabidopsis FPT* genes with that in other plant species, information on their patterns of synteny and their orthologues was retrieved from the database PLAZA (http://bioinformatics.psb.ugent.be/plaza/). Duplication analysis of *Arabidopsis FPT* genes was performed by whole genomic data of plant species obtained from PLAZA v3.0 platform.

### Phylogenetic analysis of FPT genes in Brassicaceae species

A phylogenetic tree was constructed with the aligned sequences of *FPT-*related genes from Brassicaceae species by MEGA5.2 (ref. 60) using the Maximum Likelihood. Sequences of *FPT2* were aligned by ClustalW implemented in MEGA 7.0. A phylogenetic tree of protein sequences was built by ClustalW in MEGA 7.0 with G-blocks using Gblocks 0.91b in Phylogeny.fr (http://phylogeny.lirmm.fr/phylo_cgi/one_task.cgi?task_type=gblocks)^61^.

### Data availability

Microarray data generated as part of this study has been deposited into the Gene Expression Omnibus (GEO, https://www.ncbi.nlm.nih.gov/geo/) database with accession code GSE83291. The authors declare that all other data supporting the findings of this study are available within the article and its Supplementary Information files or are available from the corresponding author on request.

## Additional information

**How to cite this article:** Tohge, T. *et al.* Characterization of a recently evolved flavonol-phenylacyltransferase gene provides signatures of natural light selection in Brassicaceae. *Nat. Commun.* 7:12399 doi: 10.1038/ncomms12399 (2016).

## Supplementary Material

Supplementary Information Supplementary Figures 1 - 17, Supplementary Tables 1 - 3 and Supplementary Note 1

Supplementary Data 1 Secondary metabolite profiling of *Arabidopsis thaliana* accession flower

Supplementary Data 2 Saiginol profiling of near-isogenic line (NIL) *Arabidopsis thaliana* lines mQTL. Samples obtained from 45 M lines (C24 background) and 69 N lines (Col-0 background) were evaluated with three independent biological replications (n=3).

Supplementary Data 3 Identification of syntenic genes located in the Brassicaceae syntenic block of *AtFPT/SCPL* genomic region. Syntenic block (defined as Block I) of *AtFPT/SCPL* genomic region, 1) *Arabidopsis thaliana* chromosome 2 (AtChr.2), were found in 2) *Arabidopsis lyrata* chromosome 4 (AlChr.4), 3) *Capsella rubella* chromosome 4 (CrChr.4), 4) *Thellungiella parvula* chromosome 4 (TpChr.4), 5) *Brassica rapa* chromosome 4 (BrChr.4) and 6) *B. rapa* chromosome 9 (BrChr.9). Syntenic blocks were found with 116 genes in *AtFPT* genomic region (AT2G22500- AT2G23470) using Plaza (http://bioinformatics.psb.ugent.be/plaza/versions/plaza_v3_dicots/). Red indicates *FPT/SCPL* related genes.

Supplementary Data 4 Identification of syntenic genes located in the intrasyntenic genomic region of *AtFPT/SCPL* genomic region in the Brassicaceae species. Syntenic genes of intrasyntenic block, *Arabidopsis thaliana* chromosome 4 (AtChr.4)(defined as Block II) of *AtFPT/SCPL* genomic region, were found in 2) *Arabidopsis lyrata* chromosome 7 (AlChr.7), 3) *Capsella rubella* chromosome 7 (CrChr.7), 4) *Thellungiella parvula* chromosome 7 (TpChr.7), 5) *Brassica rapa* chromosome 1 (BrChr.1), 6) *Brassica rapa* chromosome 3 (BrChr.3) and 7) *B. rapa* chromosome 8 (BrChr.8). Syntenic blocks were found with 132 genes in intrasyntenic genomic region (AT4G36940- AT4G38080) of *AtFPT/SCPL* region using Plaza (http://bioinformatics.psb.ugent.be/plaza/versions/plaza_v3_dicots/). Red indicates *FPT/SCPL* related genes.
