# Supplementary material for: Characterization of a recently evolved flavonol-phenylacyltransferase gene provides signatures of natural light selection in Brassicaceae
Source: Nat Commun. 2016 Aug 22;7:12399. doi: 10.1038/ncomms12399 (PMC4996938; doi:10.1038/ncomms12399)
Supplement: Supplementary Information — Supplementary Figures 1 - 17, Supplementary Tables 1 - 3 and Supplementary Note 1 [file ncomms12399-s1.pdf]

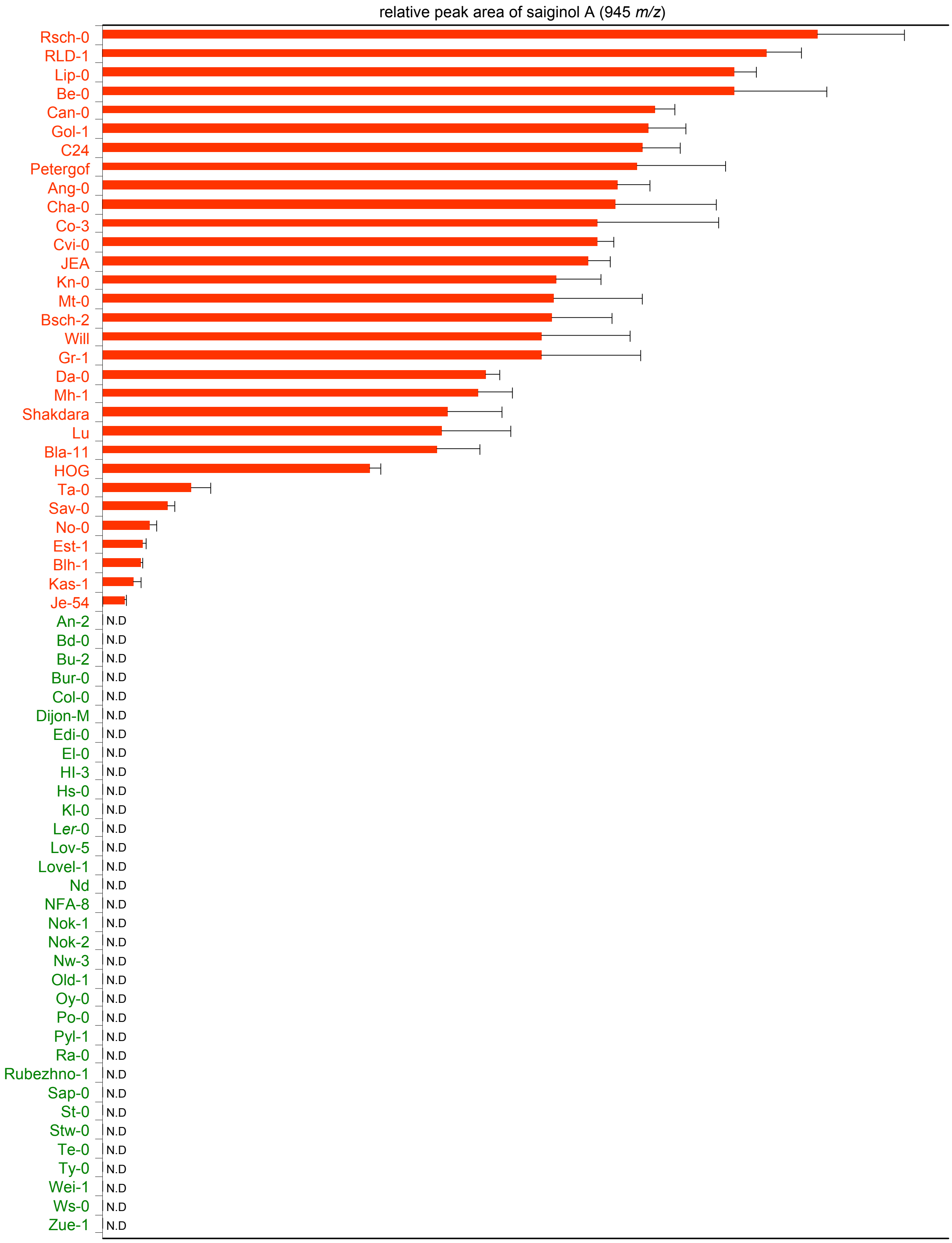

Supplementary Fig. 1 | Abundance of saiginol A in *Arabidopsis thaliana* accession flower. Content of saiginol A was presented by relative peak area (RA). A total of 64 accessions were profiled in biological triplicate (n=3). Data is presented as mean ± S.E.M.

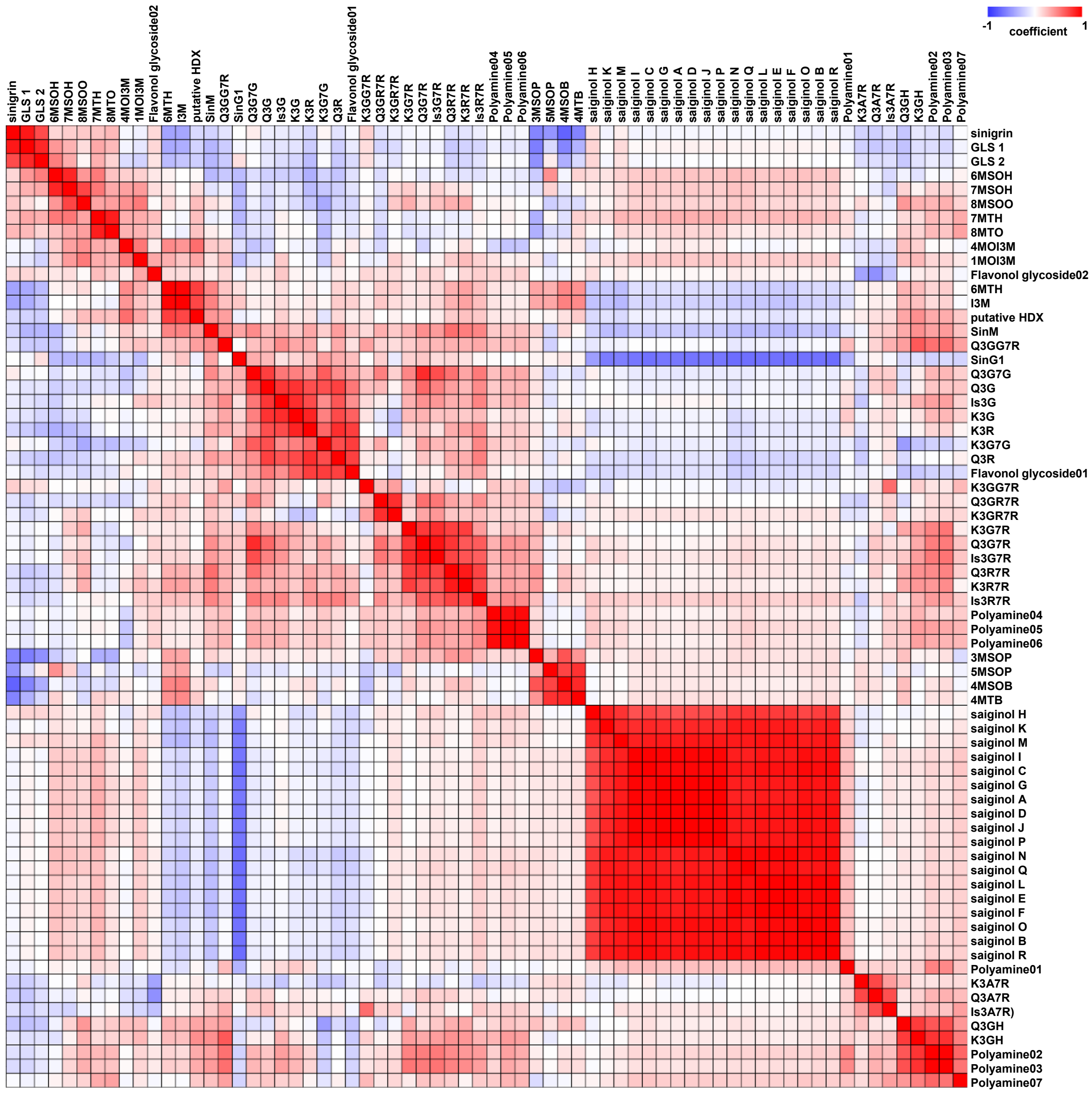

Supplementary Fig. 2 | Heatmap of coefficient correlation of metabolite contents in Arabidopsis accessions.

Hierarchical clustering (HCA) was performed by Person correlation using the values displayed on a log2 scaled relative peak area (RA) by the average of all values. Heatmap shows values displayed on coefficient values by red (1) and blue (-1).

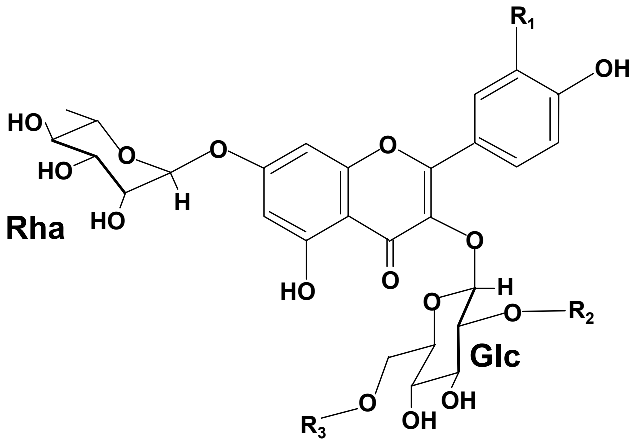

saiginol A: R_1=H, R_2=Rha, R_3=sinapoyl
saiginol B: R_1=H, R_2=Rha, R_3=caffeoyl
saiginol C: R_1=H, R_2=Rha, R_3=*p*-coumaroyl
saiginol D: R_1=H, R_2=H, R_3=sinapoyl
saiginol E: R_1=H, R_2=H, R_3=caffeoyl
saiginol F: R_1=H, R_2=H, R_3=*p*-coumaroyl
saiginol G: R_1=OH, R_2=Rha, R_3=sinapoyl
saiginol H: R_1=OH, R_2=Rha, R_3=caffeoyl
saiginol I: R_1=OH, R_2=Rha, R_3=*p*-coumaroyl
saiginol J: R_1=OH, R_2=H, R_3=sinapoyl
saiginol K: R_1=OH, R_2=H, R_3=caffeoyl
saiginol L: R_1=OH, R_2=H, R_3=*p*-coumaroyl
saiginol M: R_1=OMe, R_2=Rha, R_3=sinapoyl
saiginol N: R_1=OMe, R_2=Rha, R_3=caffeoyl
saiginol O: R_1=OMe, R_2=Rha, R_3=*p*-coumaroyl
saiginol P: R_1=OMe, R_2=H, R_3=sinapoyl
saiginol Q: R_1=OMe, R_2=H, R_3=caffeoyl
saiginol R: R_1=OMe, R_2=H, R_3=*p*-coumaroyl

Supplementary Fig. 3 | Chemical structure of saiginol A-R.
Abbreviations: Glc, glucose; Rha, rhamnose.

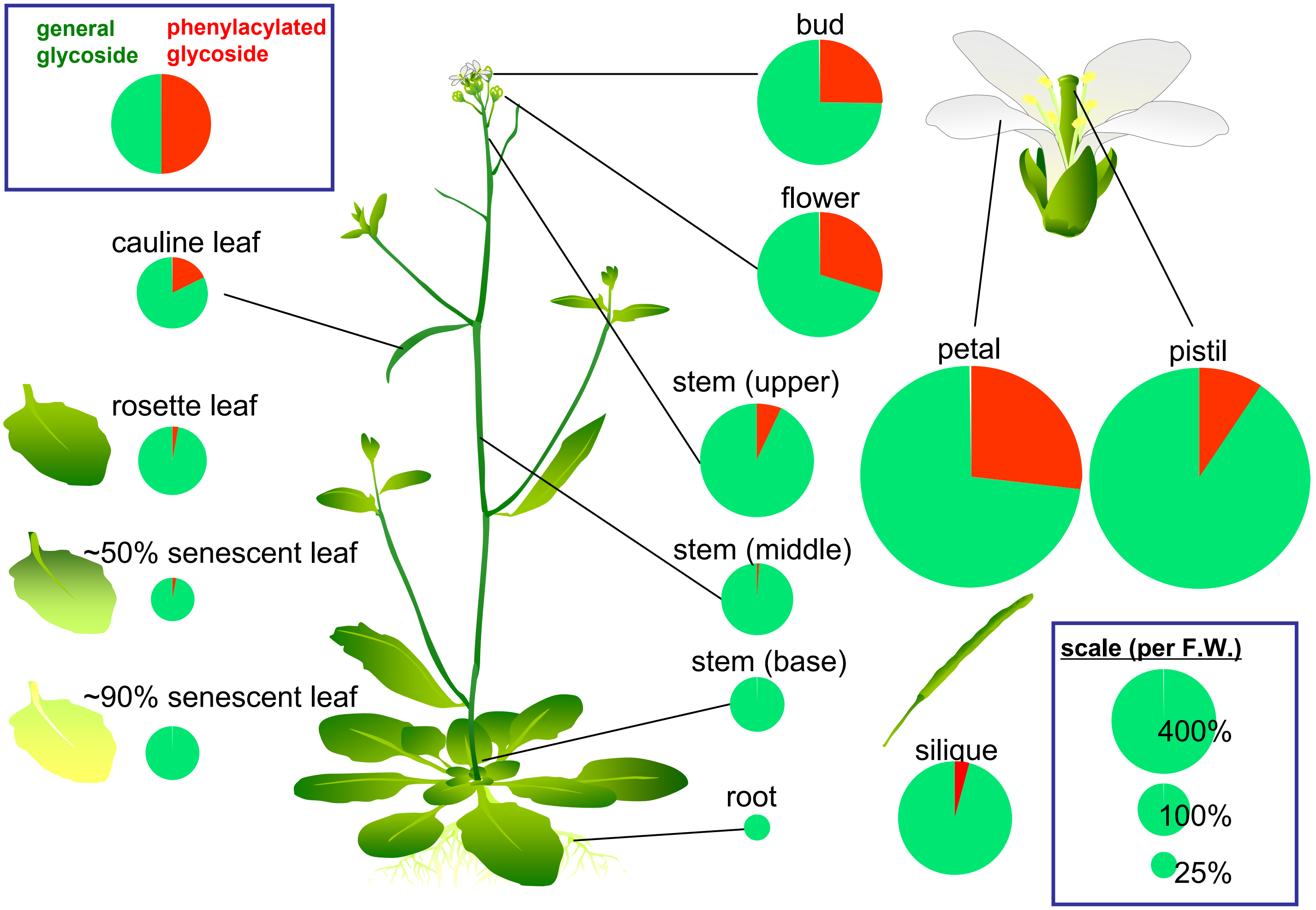

Supplementary Fig. 4 | Tissue specificity of the accumulation of saiginols.
Relative ratio of phenylacylated(red)/non-phenylacylated(green) flavonols in producer C24 accession was presented.

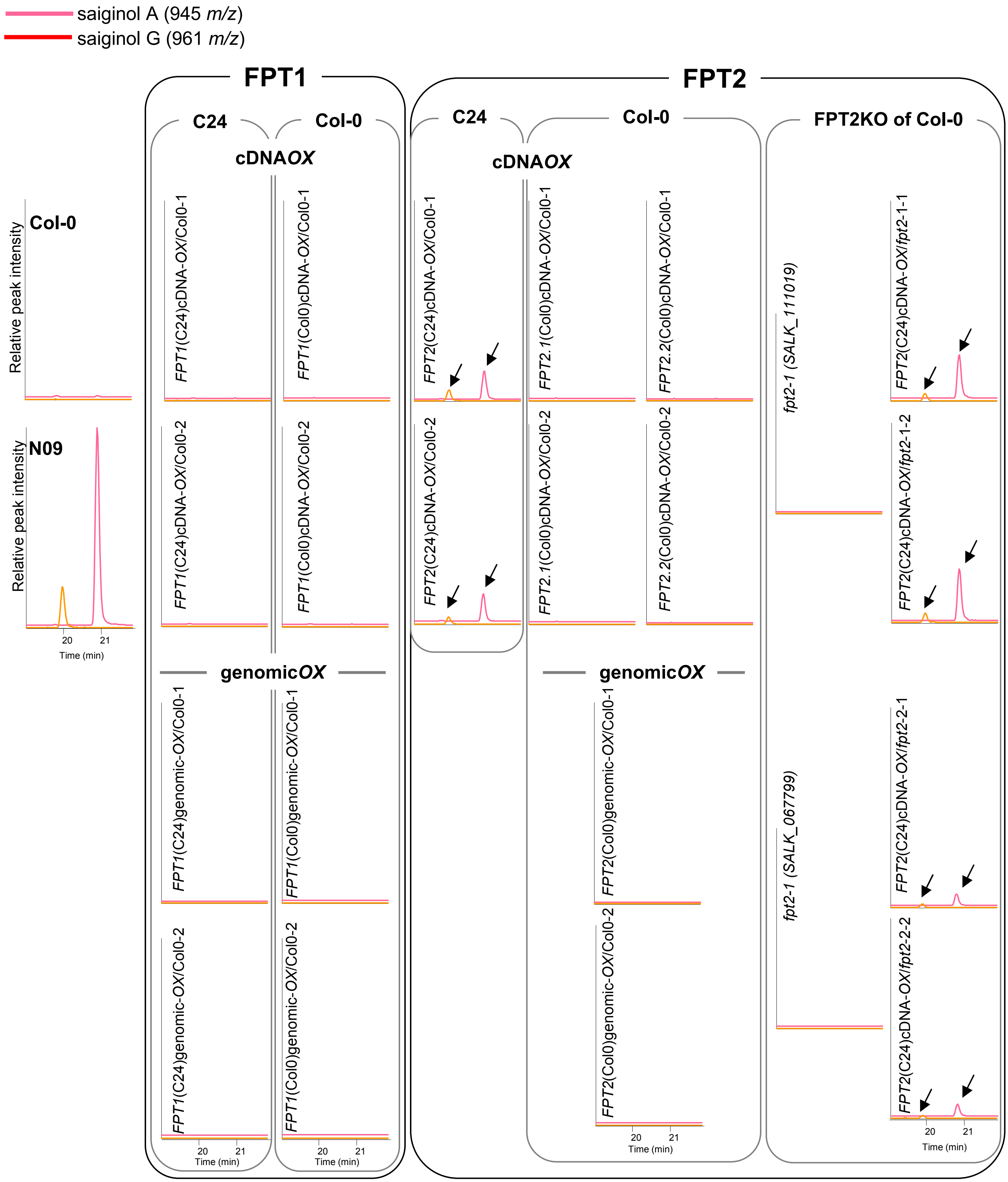

Supplementary Fig. 5 | Profiling of saiginol production in *FPT* transgenic lines. Transgenic lines which were *35S*-overexpressed of cDNA and genomic sequence of *FPT1* (At2g22920) and *FPT2* (At2g22960) cloned from Col-0 (non-producer accession) and C24 (producer accession) in Col-0 and two individual *FPT2* T-DNA knockout mutants (SALK_067799 and SALK_111019) were used for the evaluation of the function of FPT1 and FPT2.

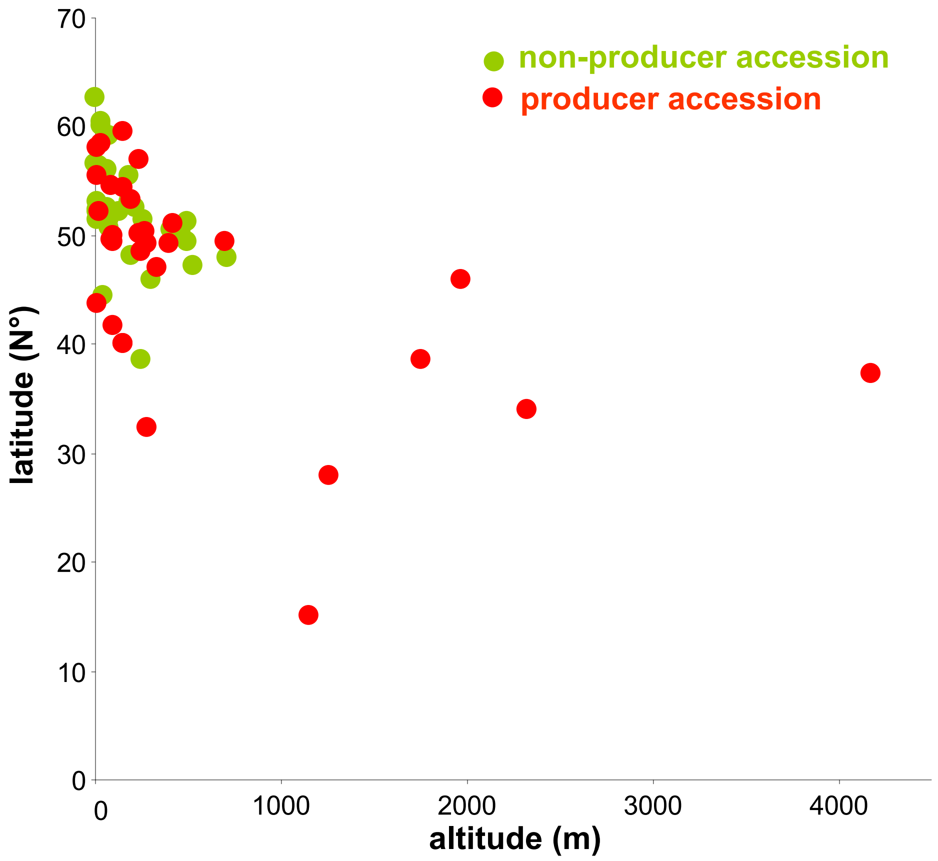

Supplementary Fig. 6 | Plots of producers and non-producers by latitude (N°) and altitude (m).

Green and orange indicates non-producing and producing accessions, respectively.

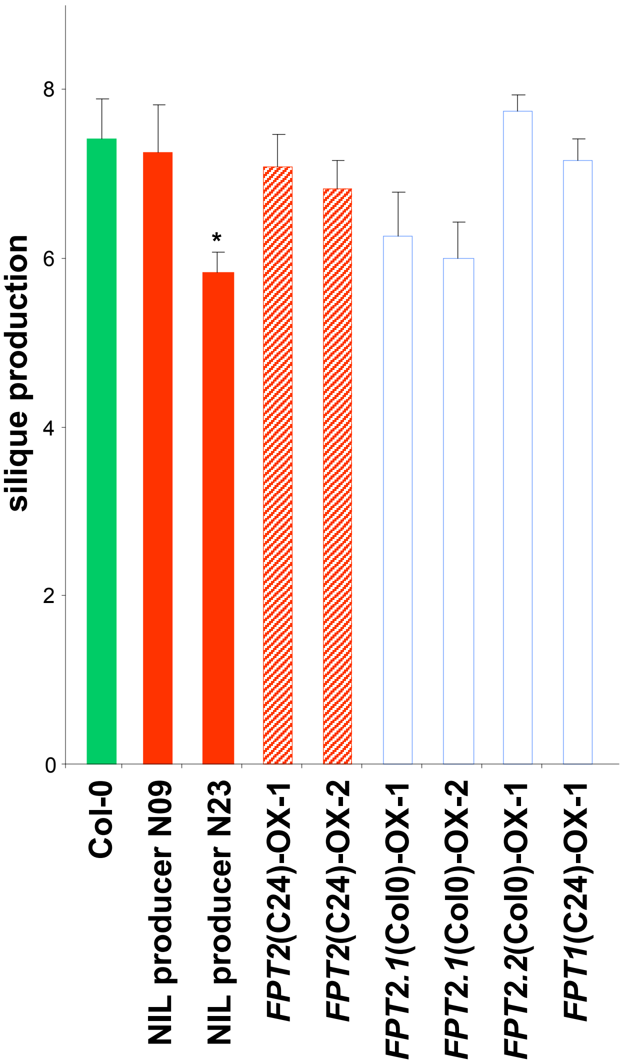

Supplementary Fig. 7 | Rate of silique production in normal light condition using the detached immature inflorescences of first bolting.

Error bars indicate the SE of 12 biological replicates. Data is presented as mean ± S.E.M. *P<0.05.

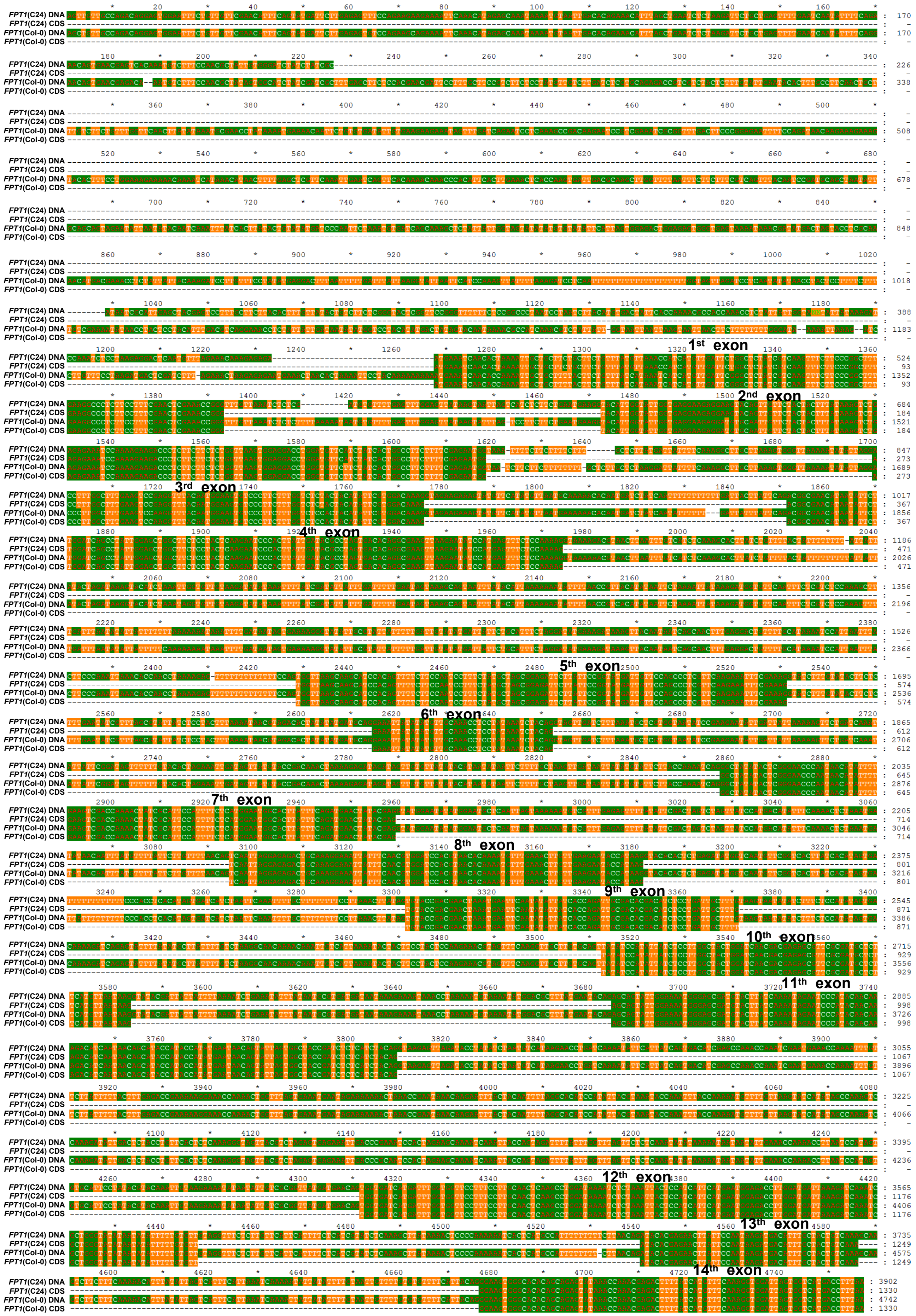

Supplementary Fig. 8 | DNA and CDS sequence mapping of *FPT1* obtained from Col-0 and C24 sequences.

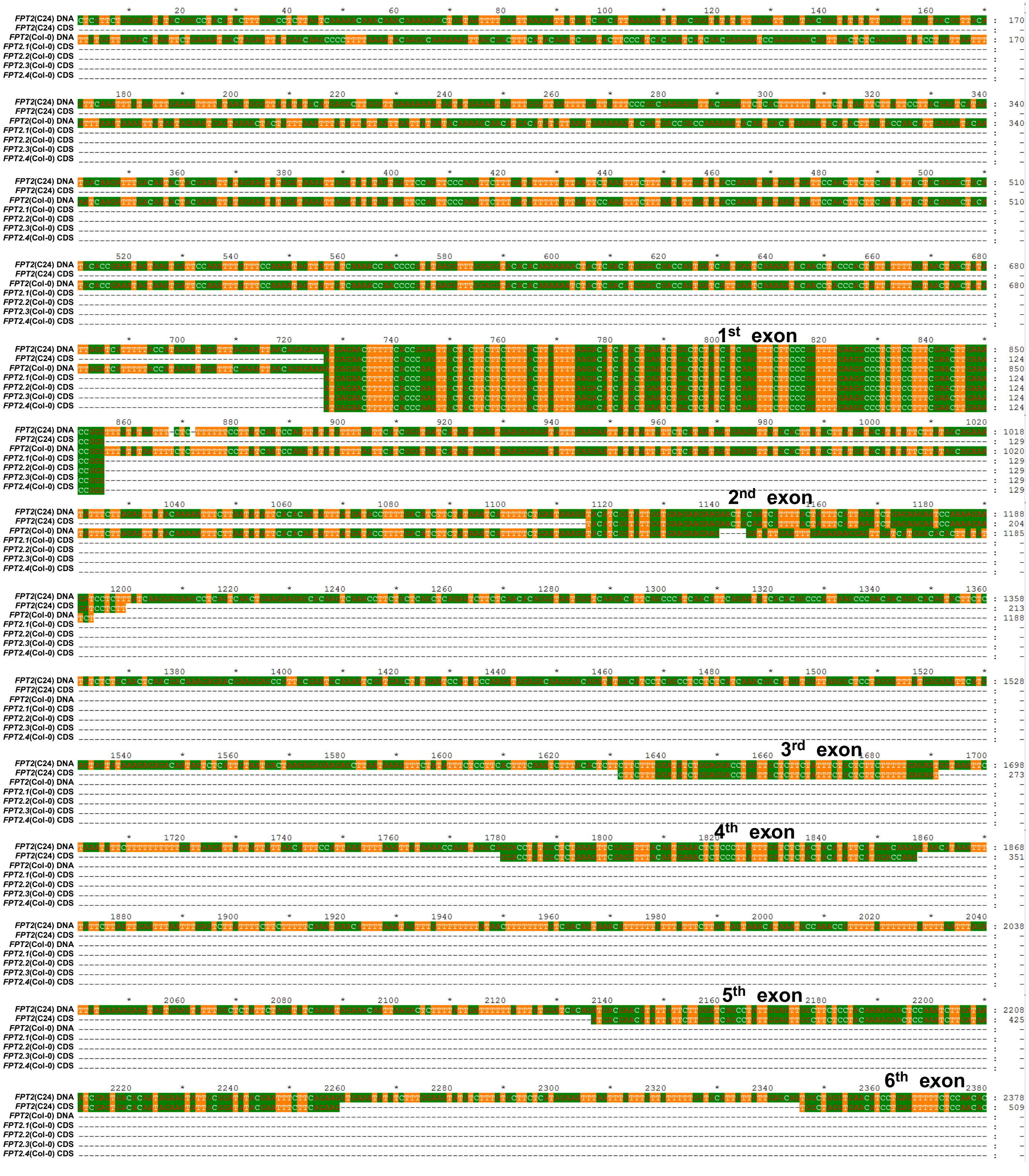

Supplementary Fig. 9a | DNA and CDS sequence mapping of *FPT2* obtained from Col-0 and C24 sequences.

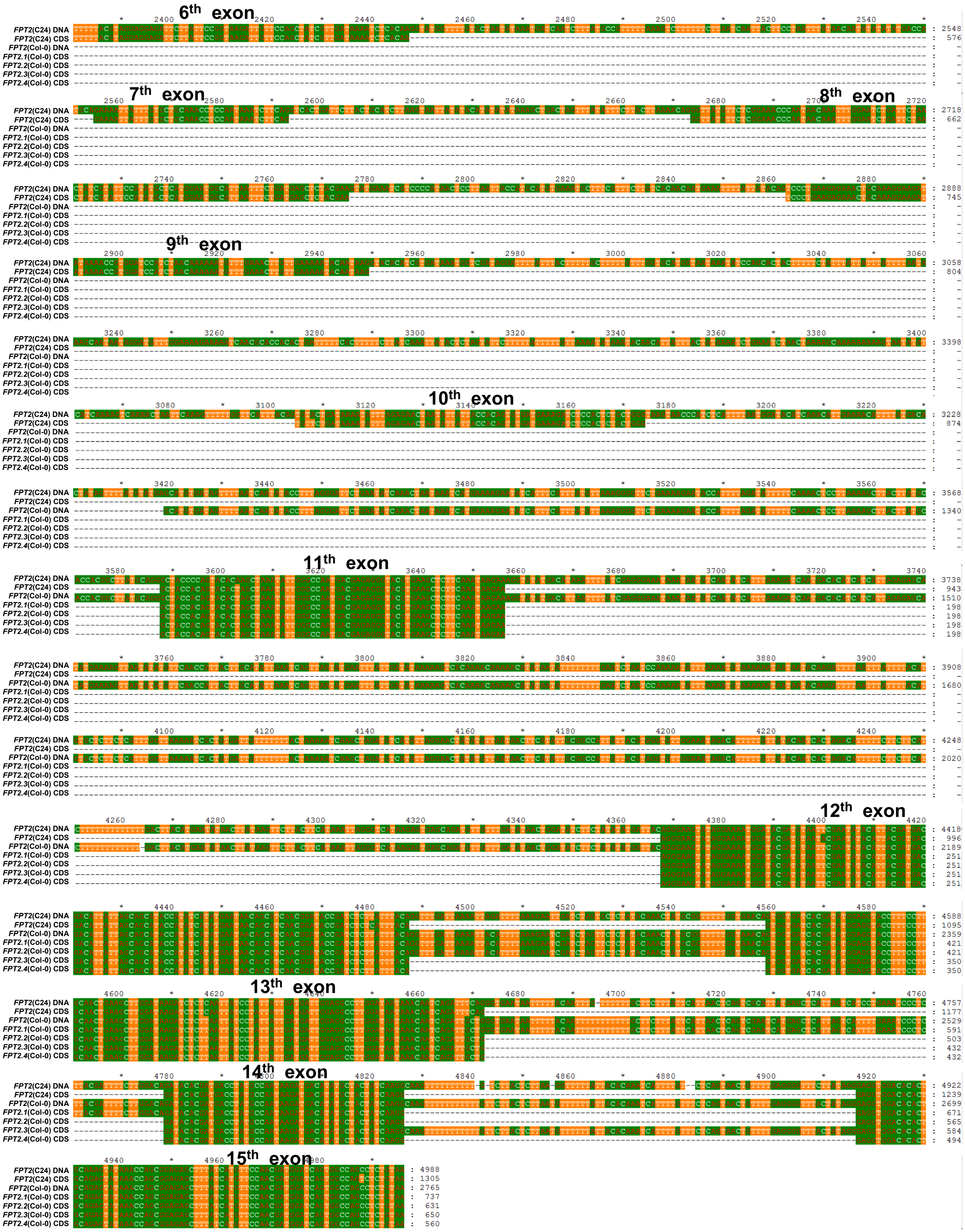

Supplementary Fig. 9b | DNA and CDS sequence mapping of *FPT2* obtained from Col-0 and C24 sequences.

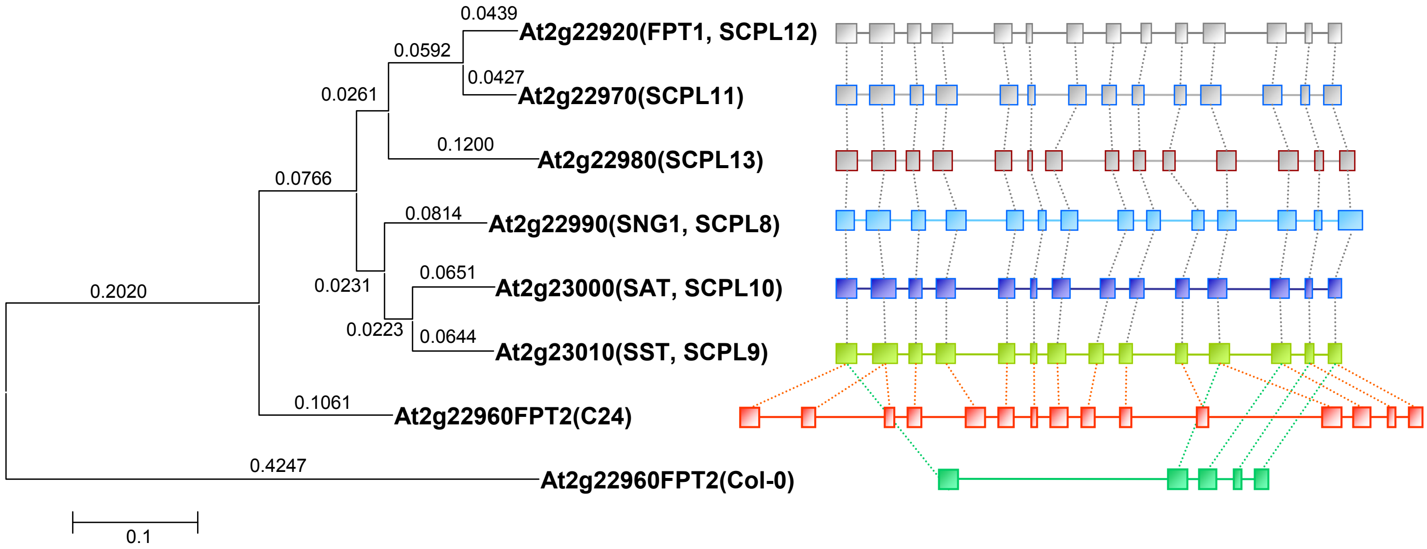

Supplementary Fig. 10 | Serine carboxypeptidase-like (*SCPL*) genes located in *FPT* duplicated region of *Arabidopsis thaliana*.

Amino acid phylogenetic tree analysis of seven *AtSCPL* genes (At2g22920, At2g22960, At2g22970, At2g22980, At2g22990, At2g23000 and At2g23010 of Col-0) in *FPT*/*SCPL* duplicated region was performed with *FPT2* (At2g22960) sequence obtained from C24 (producer accession). The phylogenetic tree was created using MEGA5.2 [60]. Scale implies nucleotide substitutions/site.

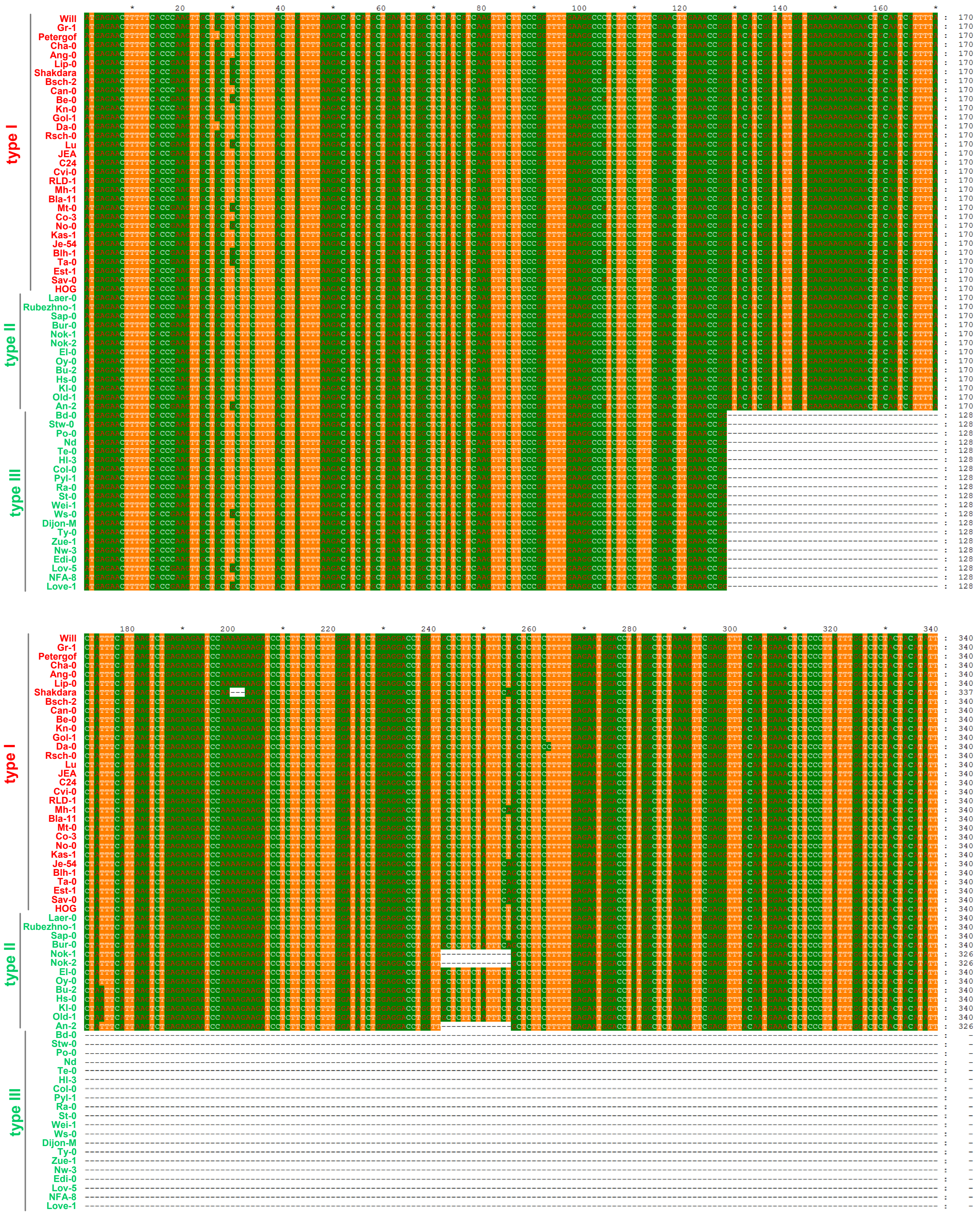

Supplementary Fig. 11a | CDS alignment of *FPT2* sequenced from 64 accessions.
Obtained sequences were also evaluated and constructed with publically available sequence (Arabidopsis 1001 genome (http://signal.salk.edu/atg1001/3.0/gebrowser.php).

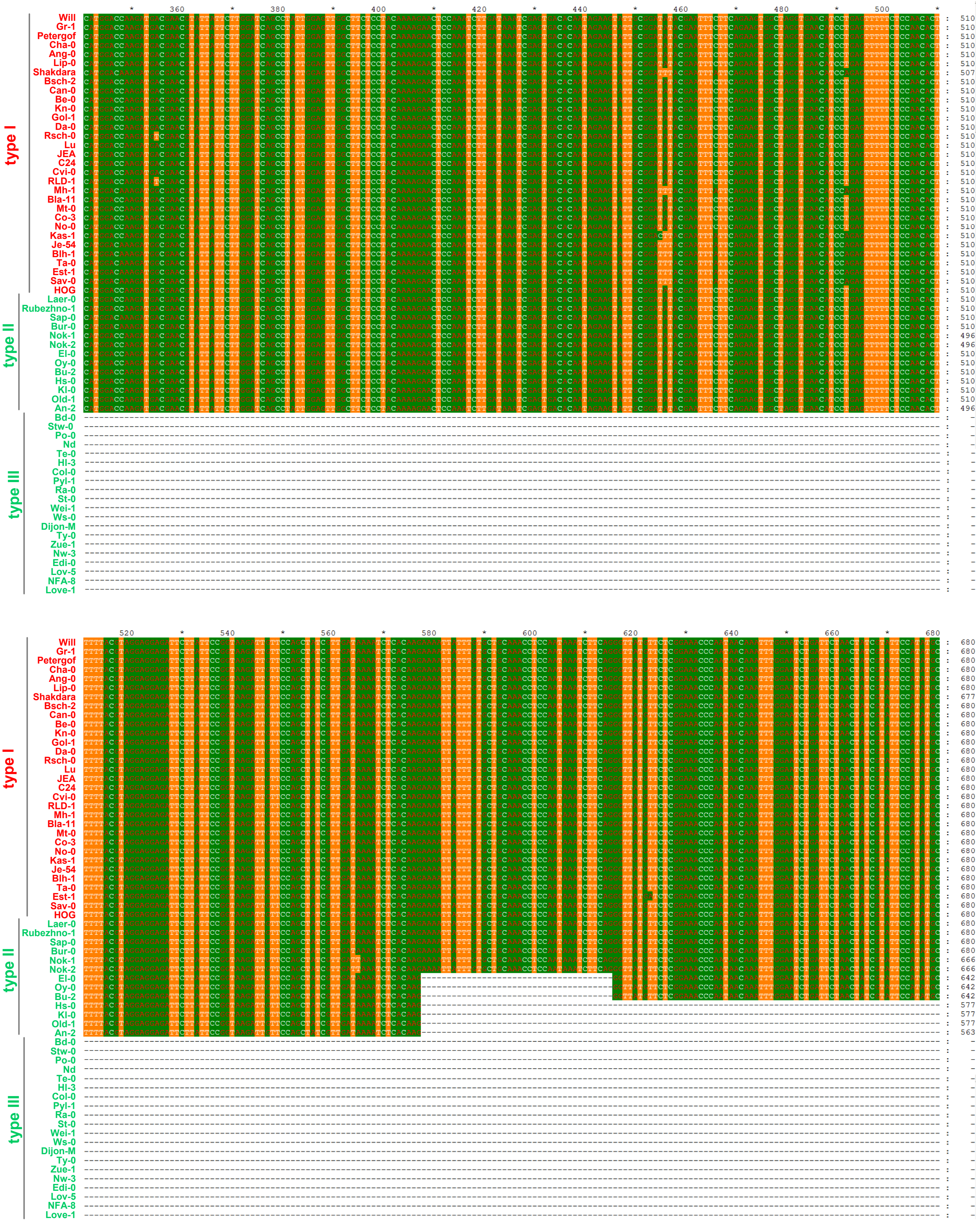

Supplementary Fig. 11b | CDS alignment of *FPT2* sequenced from 64 accessions.
Obtained sequences were also evaluated and constructed with publically available sequence (Arabidopsis 1001 genome (http://signal.salk.edu/atg1001/3.0/gebrowser.php).

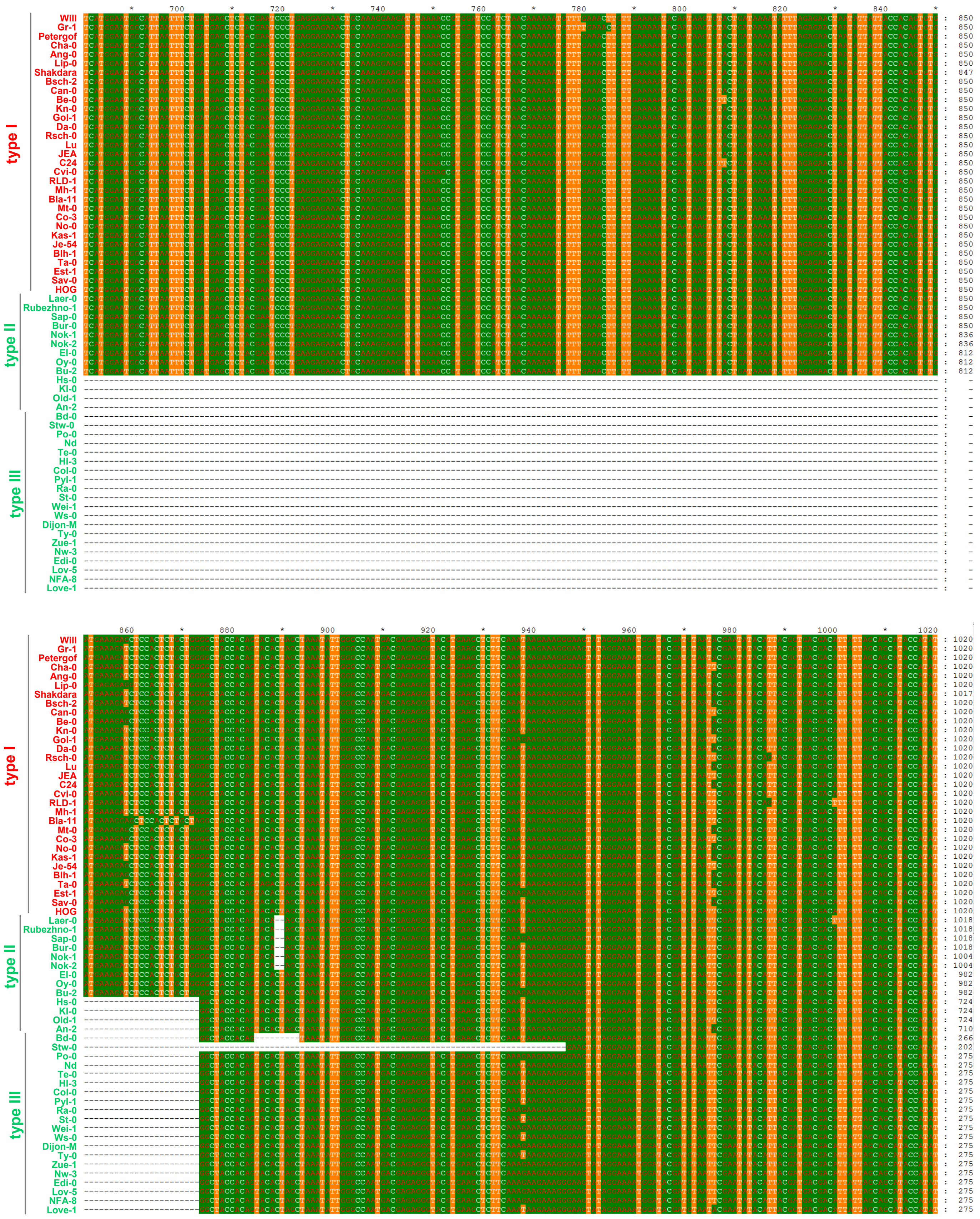

Supplementary Fig. 11c | CDS alignment of *FPT2* sequenced from 64 accessions.
Obtained sequences were also evaluated and constructed with publically available sequence (Arabidopsis 1001 genome (http://signal.salk.edu/atg1001/3.0/gebrowser.php).

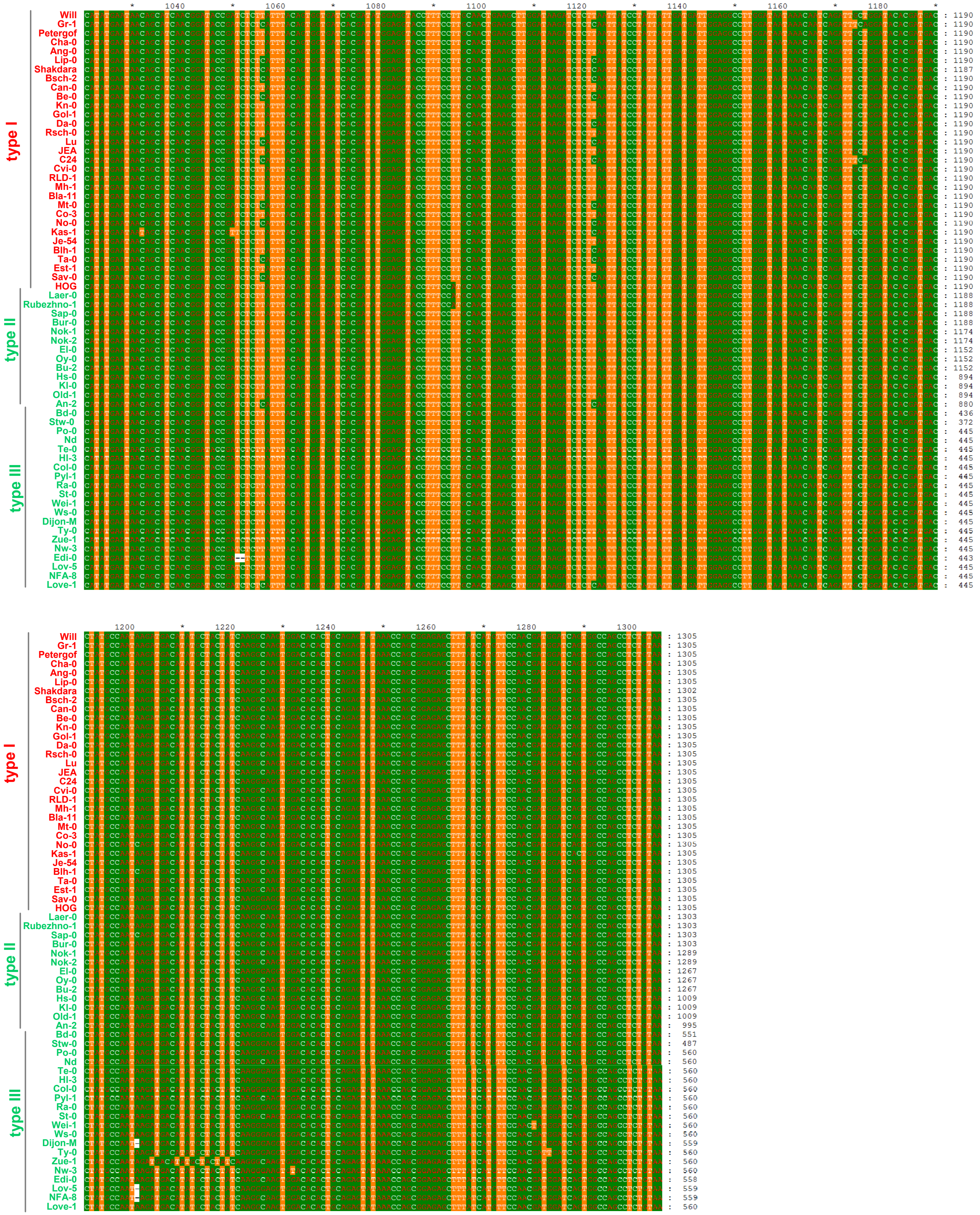

Supplementary Fig. 11d | CDS alignment of *FPT2* sequenced from 64 accessions.
Obtained sequences were also evaluated and constructed with publically available sequence (Arabidopsis 1001 genome (http://signal.salk.edu/atg1001/3.0/gebrowser.php).

	Block I			Block II		
	**chromosome**		**SCPL gene**	**chromosome**		**SCPL gene**
Arabidopsis thaliana	AtChr.2		7	AtChr. 4	inverted	0
Arabidopsis lyrata	AlChr.4		7	AlChr.7		0
Capsella rubella	CrChr.4		9	CrChr.7		0
Thellungiella parvula	TpChr.4		7	TpChr.7	inverted	0
Brassica rapa	BrChr.4		1	BrChr.1		0
	BrChr.9	inverted	1	BrChr.3	inverted	0
				BrChr.8	inverted	0
Carica papaya	-		-	-		-

Supplementary Fig. 12. Syntenic/intra-syntenic blocks of FPT genomic region found in in Brassicales plants

SCPL, serine carboxypeptidase-like family genes. "Block I" was defined as syntenic block of FPT genomic region with SCPL genes. "Block II" was defined as intrasyntenic block of FPT syntenic block which do not contain any SCPL genes.

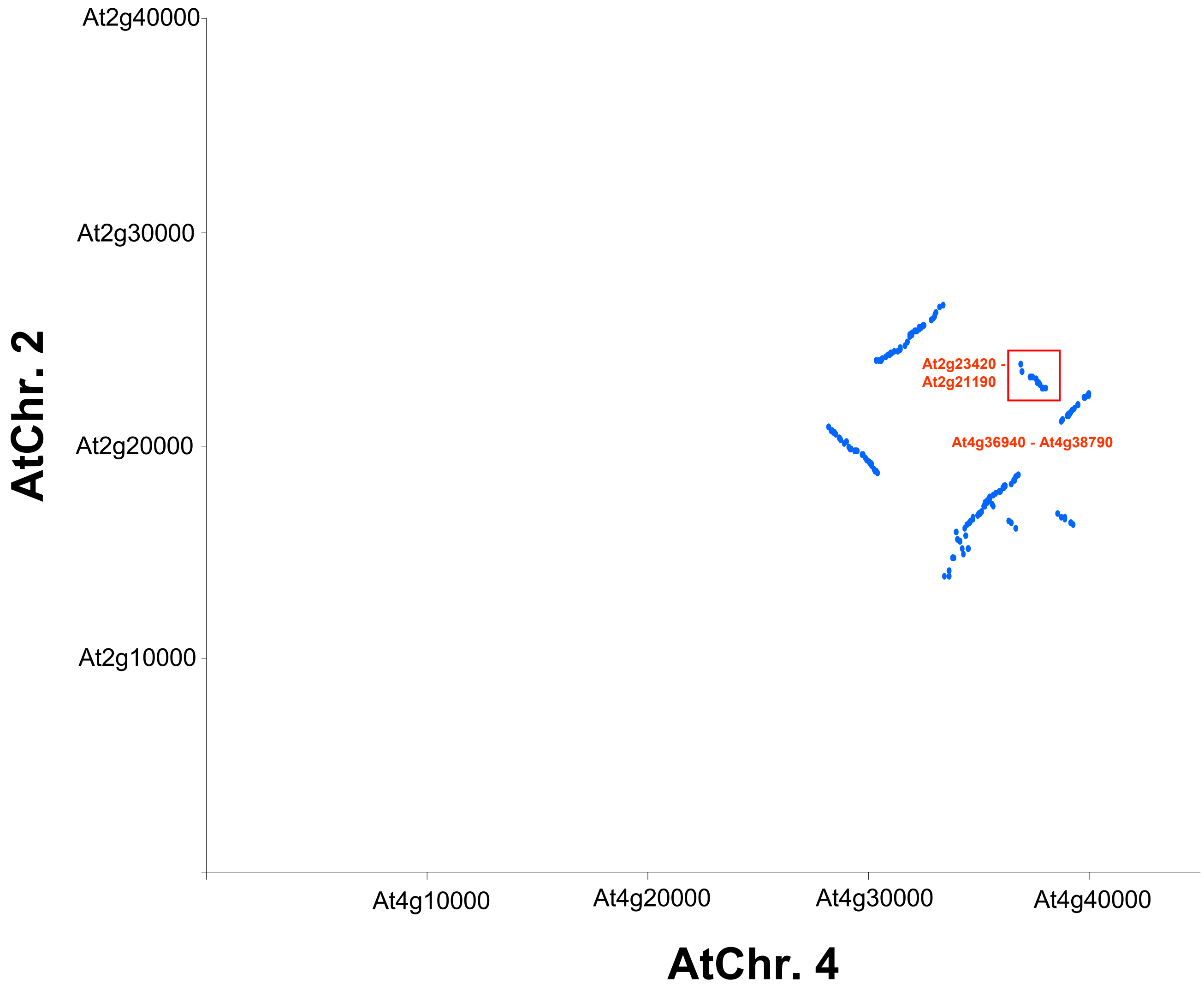

Supplementary Fig. 13 | Intrasyntenic blocks found between AtChr.2 and AtChr.4 of Arabidopsis.

FPT genes were located in inversed intrasyntenic region between At2g21190-At2g23420 and At4g38790-At4g36940 in *Arabidopsis thaliana*.

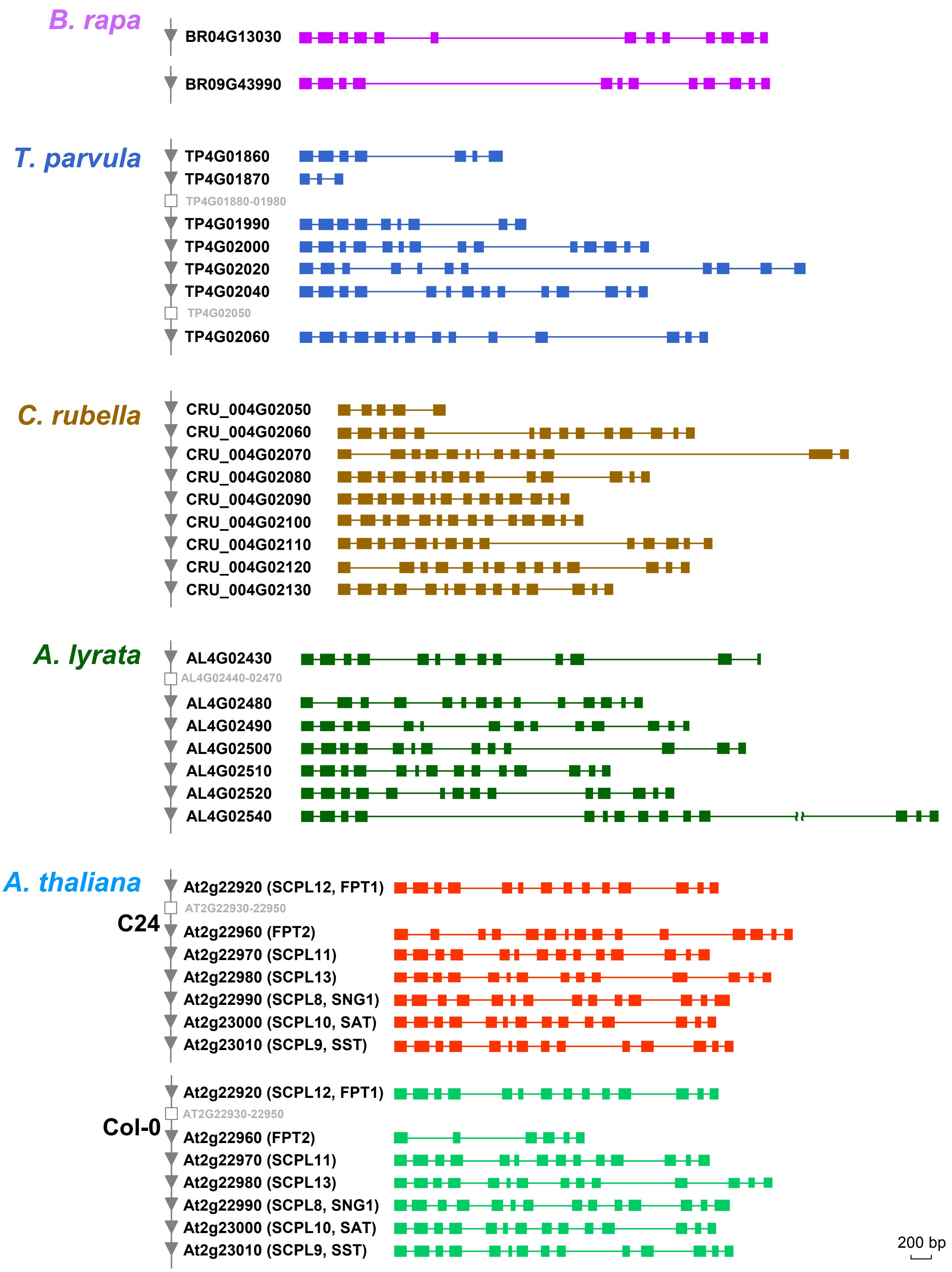

Supplementary Fig. 14 | *SCPL* genes found in *FPT*/*SCPL* gene duplicated region in Brassicaceae species.

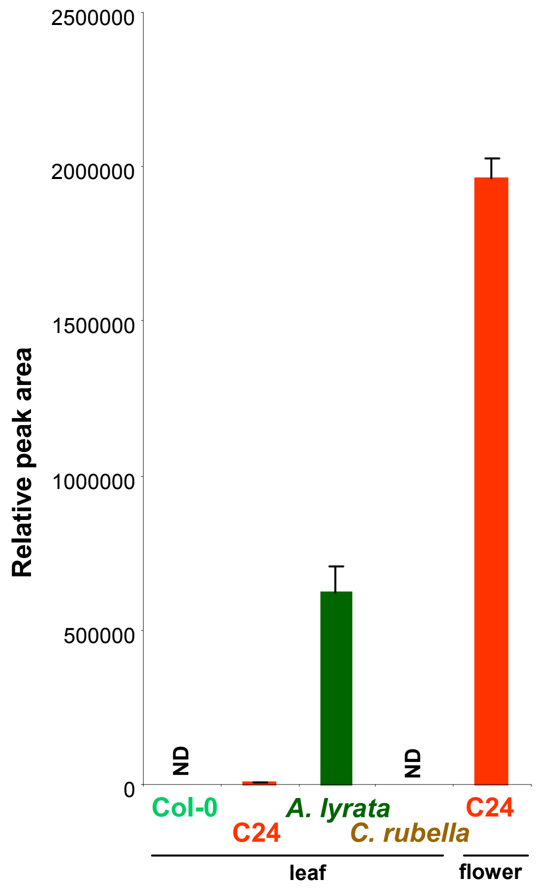

Supplementary Fig. 15 | Profiling of saiginol A in *A. thaliana*, *A. lyrata* and *C. rubella*.
LC/MS profiling of content of saiginol A in leaf of *A. thaliana* (Col-0 and C24), *A. lyrata* and *C. rubella,* and flower of *A. thaliana* C24.

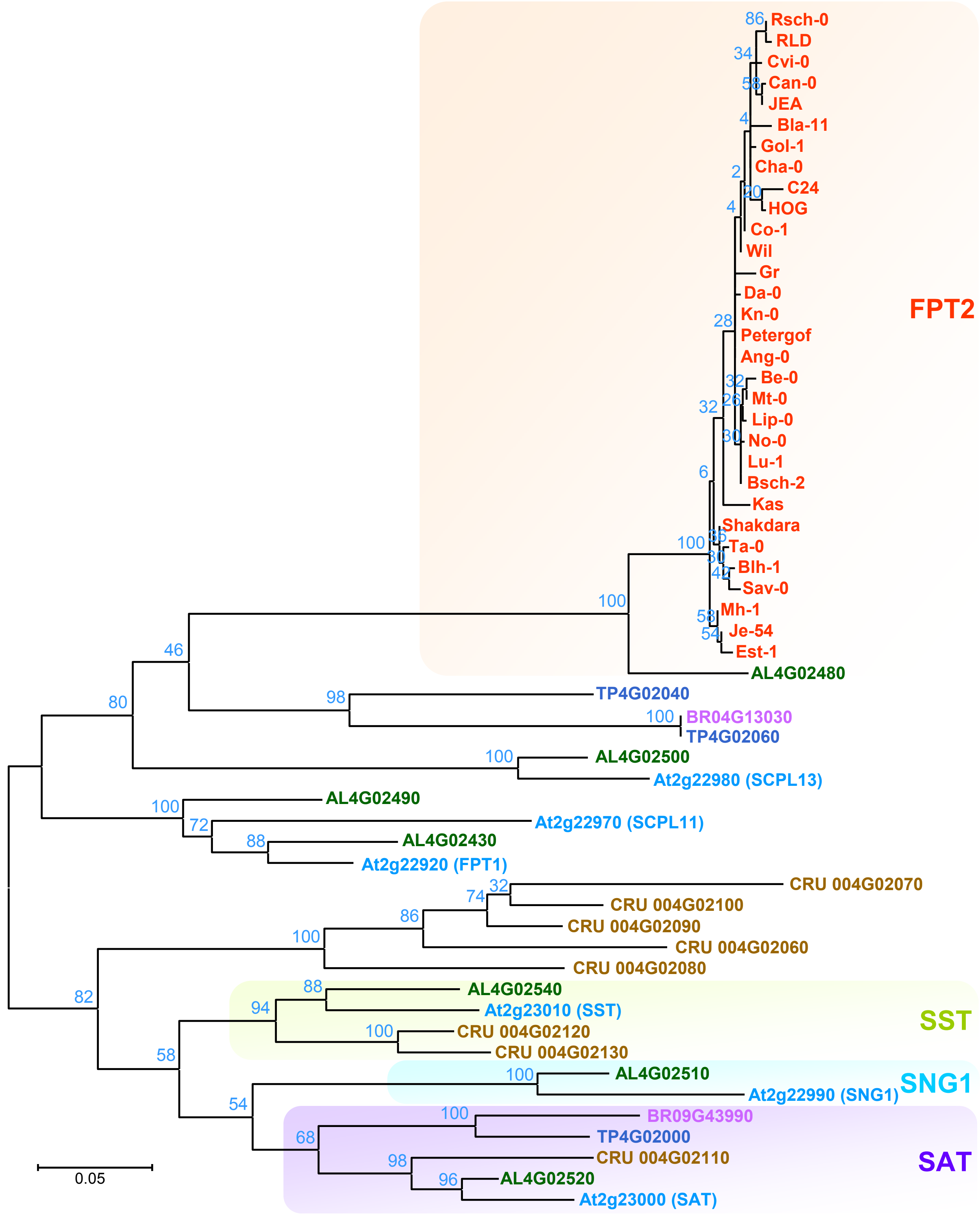

Supplementary Fig. 16 | Phylogenetic analysis using coding protein sequence of *SCPL* genes of in syntenic region of Brassicaceae species.

Phylogenetic analysis using protein sequence of *SCPL* genes of in syntenic region of Brassicaceae species presented in Figure 5d. The phylogenetic tree was created by ClustalW in MEGA7.0 [60] using G-blocks constructed by Gblocks 0.91b in Phylogeny.fr (http://phylogeny.lirmm.fr/phylo_cgi/one_task.cgi?task_type=gblocks). The genes of non-producer accessions (type II and III) and with the large deletions (CRU_004G02050, TP4G01870, TP4G01860, TP4G01990 and TP4G02020) were excluded from this analysis. Scale implies nucleotide substitutions/site.

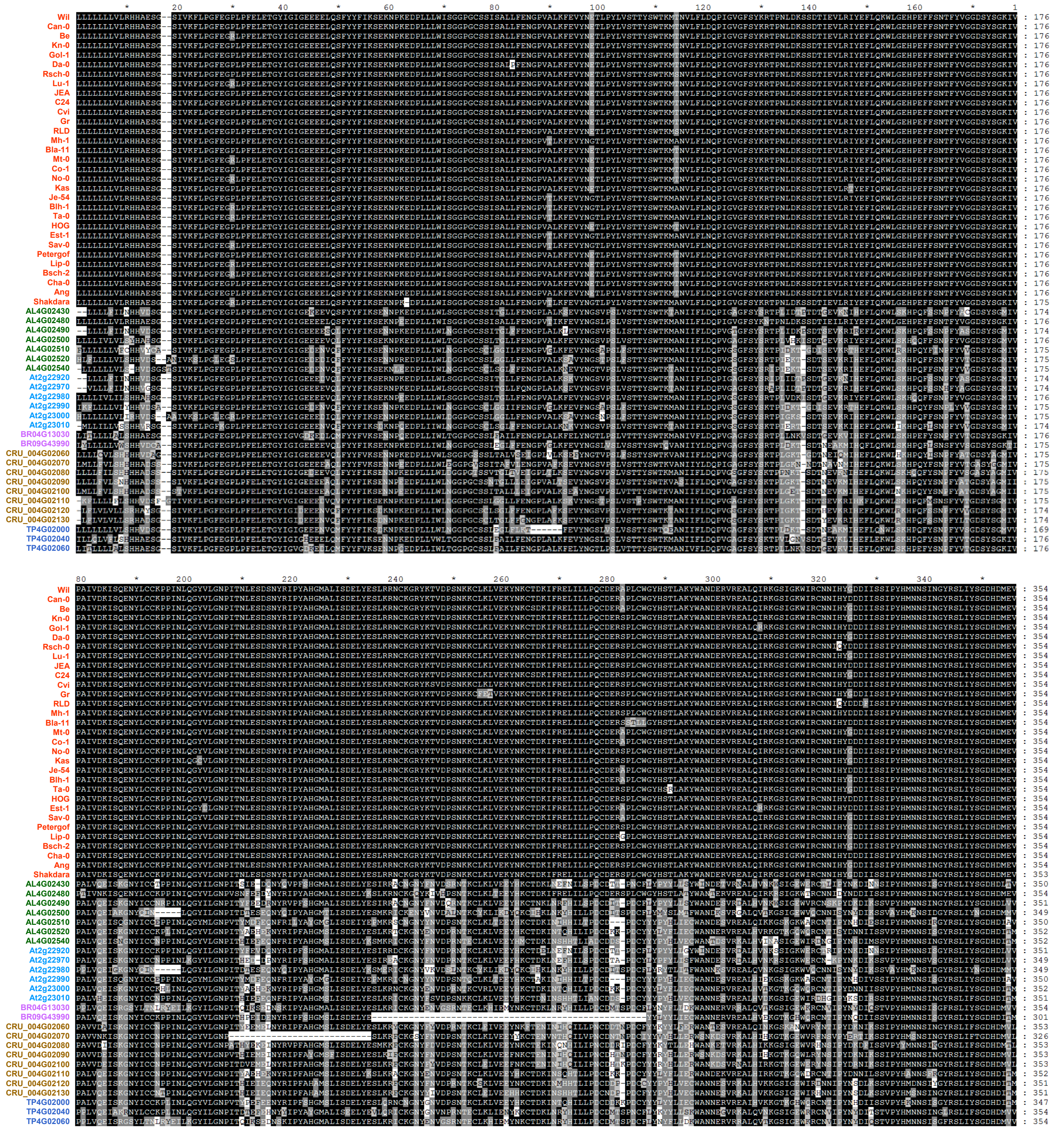

Supplementary Fig. 17a | G-block alignment using coding protein sequence of *SCPL* genes of in syntenic region of Brassicaceae species.

Sequence alignment was created by ClustalW in MEGA7.0 60 using G-blocks constructed by Gblocks 0.91b in Phylogeny.fr (http://phylogeny.lirmm.fr/phylo_cgi/one_task.cgi?task_type=gblocks).

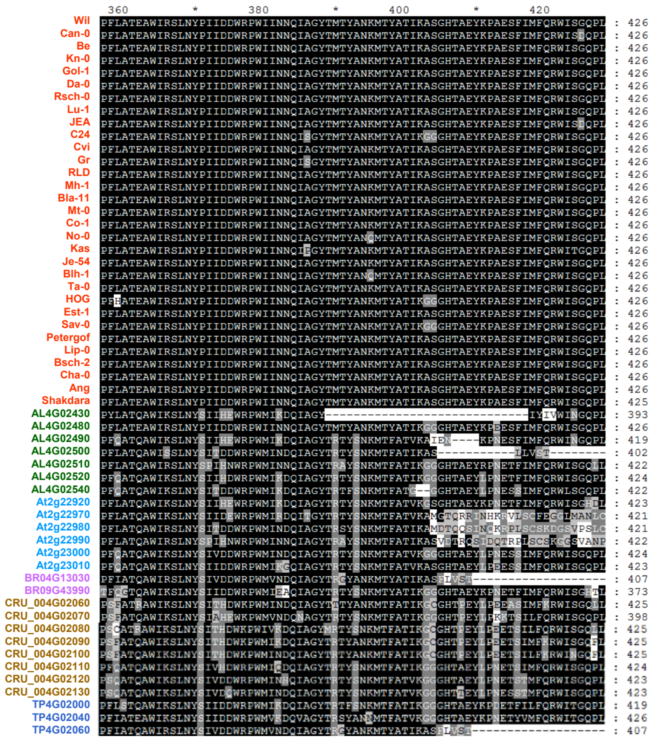

Supplementary Fig. 17b | G-block alignment using coding protein sequence of *SCPL* genes of in syntenic region of Brassicaceae species.

Sequence alignment was created by ClustalW in MEGA7.0 60 using G-blocks constructed by Gblocks 0.91b in Phylogeny.fr (http://phylogeny.lirmm.fr/phylo_cgi/one_task.cgi?task_type=gblocks).

Supplementary Table 1. Top 50 genes which are highly expressed in producing accessions.

Array Element	Locus Identifier	Chr.	FC (producer /nonproducer)	description	gene annotation
259439_at	At1g01480	**1**	**7.33**		ACS2 (1-Amino-cyclopropane-1-carboxylate synthase 2)
261020_at	At1g26390	**1**	**4.83**		FAD-binding domain-containing protein
263228_at	At1g30700	**1**	**5.89**		FAD-binding domain-containing protein
260706_at	At1g32350	**1**	**5.66**		AOX1D (ALTERNATIVE OXIDASE 1D)
261995_at	At1g33850	**1**	**4.83**		similar to N-terminal half of ribosomal protein S15
260016_at	At1g41795	**1**	**6.83**	transposable element	transposable element gene
245865_at	At1g58025	**1**	**12.95**		unknown protein
257500_s_at	At1g73300, At5g36180	**1**	**4.96**	SCPL	SCPL2 (serine carboxypeptidase-like 2)
260225_at	At1g74590	**1**	**13.23**		ATGSTU10 (Arabidopsis thaliana Glutathione S-transferase (class tau) 10)
267479_at	At2g02690	**2**	**4.84**		unknown protein
263768_x_at	At2g06330	**2**	**4.90**	transposable element	transposable element gene
266385_at	At2g14610	**2**	**5.64**		PR1 (PATHOGENESIS-RELATED GENE 1)
263071_at	At2g17490	**2**	**5.81**	transposable element	transposable element gene
265587_at	At2g19980	**2**	**7.70**		allergen V5/Tpx-1-related family protein
266827_at	At2g22920	**2**	**17.73**	FPT1, SCPL12	SCPL12; serine carboxypeptidase
267253_at	At2g22960	**2**	**6.68**	FPT2, pseudo SCPL	serine carboxypeptidase S10 family protein
263948_at	At2g35980	**2**	**6.27**		YLS9 (YELLOW-LEAF-SPECIFIC GENE 9)
265877_at	At2g42380	**2**	**6.72**		bZIP transcription factor family protein
260568_at	At2g43570	**2**	**6.36**		chitinase, putative
266930_at	At2g45930	**2**	**4.81**		unknown protein
257364_at	At2g45940	**2**	**6.13**		unknown protein
266607_at	At2g46300	**2**	**4.93**		unknown protein
259206_at	At3g09040	**3**	**4.99**		pentatricopeptide (PPR) repeat-containing protein
256933_at	At3g22600	**3**	**4.87**		protease inhibitor/seed storage/lipid transfer protein (LTP) family protein
256859_at	At3g22940	**3**	**4.96**		F-box family protein-related
258028_at	At3g27473	**3**	**5.56**		DC1 domain-containing protein
257774_at	At3g29250	**3**	**5.54**		oxidoreductase
245222_at	At3g29787	**3**	**36.63**	transposable element	transposable element gene
256940_at	At3g30720	**3**	**8.38**		unknown protein
256610_at	At3g30845	**3**	**4.90**		unknown protein
256694_at	At3g32080	**3**	**6.95**	transposable element	transposable element gene
256643_at	At3g32290	**3**	**8.08**	transposable element	unknown protein similar to putative retroelement pol polyprotein
252775_s_at	At3g42930, At5g30545	**3**	**8.33**	transposable element	transposable element gene
251625_at	At3g57260	**3**	**4.79**		BGL2 (PATHOGENESIS-RELATED PROTEIN 2)
251344_at	At3g60920	**3**	**8.80**		beige/BEACH domain-containing protein
255437_at	At4g03060	**4**	**10.73**		AOP2 (ALKENYL HYDROXALKYL PRODUCING 2), glucosinolate
255375_at	At4g03780	**4**	**4.88**	transposable element	transposable element gene
255340_at	At4g04490	**4**	**6.29**		protein kinase family protein
255146_at	At4g08450	**4**	**4.95**		disease resistance protein (TIR-NBS-LRR class), putative
245393_at	At4g16260	**4**	**10.01**		glycosyl hydrolase family 17 protein
253707_at	At4g29200	**4**	**18.86**		beta-galactosidase
253505_at	At4g31970	**4**	**12.08**		CYP82C2 (cytochrome P450)
246252_s_at	At4g37070, At4g37060	**4**	**5.62**		PLA IVA/PLP1; nutrient reservoir
253044_at	At4g37290	**4**	**6.08**		unknown protein
250983_at	At5g02780	**5**	**8.60**		In2-1 protein, putative
256136_s_at	At5g13205, At1g58140, At1g48710, At1g48720, At3g59720, At1g11265, At1g21945, At3g61330	**5**	**5.25**	transposable element	transposable element gene
246927_s_at	At5g25260, At5g25250	**5**	**8.12**		unknown protein

246826_at	At5g26310	**5**	**4.84**	UGT72E3; UDP-glycosyltransferase/ coniferyl-alcohol glucosyltransferase
249599_at	At5g37990	**5**	**4.91**	S-adenosylmethionine-dependent methyltransferase
249465_at	At5g39720	**5**	**6.59**	AIG2L (AVIRULENCE INDUCED GENE 2 LIKE PROTEIN)

Supplementary Table 2. The site of origin of Arabidopsis accessions used in this study.

accession name	saiginol production	country (Sulpice et al., 2007)	city	country (1001 genomes project)	altitude literature	altitude SRTM	latitude	longitude	UV-B irradiation (J/m^2)
An-2	non-producer	Belgium	Antwerpen	Netherlands	1-100	9.00	51.50	4.50	18727
Ang-0	producer	Belgium	Angleur (Prov. de Liege)	Belgium	1-100	270.44	50.37	5.35	17935
Bd-0	non-producer	Germany	Berlin/Dahlem	Germany	1-100	47.25	52.30	13.24	17762
Be-0	producer	Germany	Bensheim/Bergstr.	Germany	100-200	99.39	49.41	8.37	18301
Bla-11	producer	Spain	Blanes/Gerona	Spain	1-100	95.48	41.70	2.79	29140
Blh-1	producer		Bulhary	Austria	100-200	248.38	48.49	16.45	19019
Bsch-2	producer	Germany	Buchschlag/FFM	Germany	1-100	95.85	50.01	8.40	17615
Bu-2	non-producer	Germany	Burghaun/Rhon	Germany	260	465.38	50.42	9.43	17054
Bur-0	non-producer	Ireland	Burren (Eire)	ireland	1-100	186.18	53.07	-9.04	16561
C24	producer	Portugal	Lousa	Portugal	100-200	146.86	40.12	-8.25	30139
Can-0	producer	Canary Island	Las Palmas de Gran Canaria	Morocco	1200-1300	1260.00*	28.00	-15.30	37817
Cha-0	producer	Switzerland	Champex	Switzerland	1500-1600	1973.75	46.02	7.07	27714
Co-3	producer	Portugal	Coimbra	Portugal	100-200	146.86	40.12	-8.25	30139
Col-0	non-producer	USA	Columbia	USA	1-100	249.43	38.50	-92.50	not available
Cvi-0	producer	Cape Verde Islands	Cape Verdi Islands	Senegal	1100-1200	1150*	15.00	-24.40	41001
Da-0	producer	Germany	Darmstadt	Germany	100-200	89.07	49.52	8.38	18074
Dijon-M	non-producer	Unknown		Russia	100-200	186.31	55.45	37.35	17340
Edi-0	non-producer	United Kingdom	Edinburgh	UK	1-100	64.82	55.95	-3.18	14661
El-0	non-producer	Germany	Ellershausen	Germany	100-200	492.74	51.31	9.40	17106
Est-1	producer	Estland	Estland	Estonia	100-200	29.11	58.35	25.00	17338
Gol-1	producer		Scotland Golspie	UK	1-100	6.73	57.97	-3.97	14871
Gr-1	producer		Graz, Austria	Austria	1-100	332.00	47.00	15.50	21147
HI-3	non-producer	Germany	Holtensen	Germany	200-300	260.26	51.50	9.50	17225
HOG	producer		Tadjikistan, Khodga-Obi-Garm	Tajikistan	1700-1800	1750.00	38.55	68.47	not available
Hs-0	non-producer	Germany	Hannover/Stroehen	Germany	1-100	39.13	52.50	9.50	16237
Je-54	producer	Czechoslovakia		Czech republic	250	278.97	49.30	17.00	18277
JEA	producer	France	St Jean Cap Ferrat (06230)	France	1-100	10.83	43.69	7.33	28119
Kas-1	producer		Kashimir	Jammu and Kashmir	1580	2324.00	34.00	74.00	not available
Kl-0	non-producer	Germany	Koeln	Germany	1-100	414.06	50.55	6.57	17859
Kn-0	producer	Lithuania	Kaunas	Lithuania	1-100	87.00	54.54	23.54	20136
L*er*-0	non-producer		Landsberg	Poland	1-100	66.48	52.50	15.50	17431
Lip-0	producer	Poland	Lipowiec/Chrzanow	Poland	400-500	240.08	50.09	19.24	16982
Lov-5	non-producer	Sweden	Lovvik (harnosand Area)	Sweden	1-100	2.60	62.63	17.94	16450
Lovel-1	non-producer			Denmark	1-100	2.96	56.57	9.48	19796
Lu	producer	Sweden	Lund	Sweden	1-100	13.10	55.40	13.13	18274
Mh-1	producer	Poland	Muehen (OstPr)	Poland	100-200	193.19	53.31	20.12	17758
Mt-0	producer	Libya	Martuba/Cyrenaika	Libya	100-200	283.05	32.34	22.46	35451
Nd	non-producer	Germany	Golm-Potsdam	Germany	40	49.05	52.24	12.58	17668
NFA-8	non-producer	United Kingdom	Ascot (England)	UK	1-100	79.90	51.41	-0.70	16229
No-0	producer	Germany		Germany	400	417.73	51.00	9.00	16960
Nok-2	non-producer	Netherlands	Noordwijk	Netherlands	1-100	7.90	52.23	4.26	20483
Nok-1	non-producer	Netherlands	Noordwijk	Netherlands	1-100	7.90	52.37	4.47	20874

Nw-3	non-producer	Germany	Neuweilnau	Germany	100-200	457.83	50.19	8.25	17202
Old-1	non-producer	Germany	Oldenburg	Germany	1-100	9.27	53.10	8.10	17950
Oy-0	non-producer	Norway	Oystese	Norway	1-100	31.04	60.38	6.20	12640
Petergof	producer	Russia	Petergof	Russia	100-200	153.31	59.53	29.53	17487
Po-0	non-producer	Germany	Poppelsdorf	Germany	1-100	72.12	50.72	7.09	16940
Pyl-1	non-producer	France	Le Pyla (33115)	France	1-100	45.35	44.39	-1.10	25981
Ra-0	non-producer	France	Randan, Puy-de-Dome	France	400-500	305.82	46.00	3.50	23665
RLD-1	producer	Netherlands		Netherlands	1-100	17.76	52.15	5.30	18755
Rsch-0	producer	Russia	Rschew/Starize	Russia	100-200	231.67	57.00	33.60	16520
Rubezhno-1	non-producer	Ukraine	Rubezhnoe	Ukraine		189.21	48.13	37.19	22264
Sap-0	non-producer	Czechoslovakia	Slapy	Czech republic	400-500	494.61	49.49	14.24	19126
Sav-0	producer	Czechoslovakia	Slavice	Germany	500-600	701.00	49.49	12.56	18612
Shakdara	producer	Tadjikistan	Shakdara River (Pamir)	Afghanistan	3300-3400	4178.09	37.29	71.30	not available
St-0	non-producer	Sweden	Stockholm	Sweden	1-100	76.69	59.19	18.03	17903
Stw-0	non-producer	Russia	Stobowa/Orel	Russia	100-200	217.00	52.57	36.04	19751
Ta-0	producer	Czechoslovakia	Tabor	Czech republic	400-500	398.20	49.25	14.40	18996
Te-0	non-producer	Finland	Tenala	Finland	1-100	30.91	60.04	23.18	17393
Ty-0	non-producer		Taynuilt	UK		19.26	56.43	-5.24	13523
Wei-1	non-producer	Switzerland	Weiningen	Switzerland	400-500	529.38	47.25	8.25	21284
Will	producer	Lithuania	Vilnius	Lithuania	100-200	147.89	54.41	25.19	19860
Ws-0	non-producer	Belarus	Wassilewskija	Belarus	100-200	129.00	52.13	30.38	20249
Zue-1	non-producer		ZURICH	Germany		707.60	48.00	8.50	20649

Altitude are shown by data from literatures (Sulpice et al., 2007) and 1001 genomes project (http://1001genomes.org/), and calculated by Shuttle Radar Topography Mission (SRTM, http://www2.jpl.nasa.gov/srtm/). * indicates altitude from 1001 genomes project (http://1001genomes.org/). UV-B irradiation (J/m2) averaged of daily UV-B irradiance during 1985 and 2005 estimated from satellite data (Soda (http://www.soda-is.com/eng/index.html)).

Supplementary Table 3. Primers used for sequencing and cloning of FPT1 and FPT2.

Primers were designed using NCBI blast primer (http://www.ncbi.nlm.nih.gov/tools/primer-blast/)

primer name	sequence	length
DNA-cloning of At2g22920 from C24 and Col0		
cDNA_At2g22920_FW3	ATGAAATCAACACCAAAAT	19
cDNA_At2g22920_REV1	ATTTAGGTAAGGTCATTCAAGTA	23
cDNA-cloning of At2g22960 from C24 and Col0		
cDNA_At2g22960_FW1	ATGAGAACTTTTTCACCCAAGTT	23
cDNA_At2g22960_REV1	TCATCATCTCTTATTATTACAGA	23
genomic sequence cloning from At2g22920 from C24 and Col-0		
BP-20-FW	GGGGACAAGTTTGTACAAAAAAGCAGGCTCCACCATGAAATCAACACCAAAATTGCT	57
BP-20-REV	GGGGACCACTTTGTACAAGAAAGCTGGGTCGTGCCCACTTCCCTGTAATG	50
genomicDNA-cloning from At2g22960 from Col0		
BP-P64-60-FW	GGGGACAAGTTTGTACAAAAAAGCAGGCTCCACCATGAGAACTTTTTCACCCAAGTT	57
BP-P66-60-REV	GGGGACCACTTTGTACAAGAAAGCTGGGTCTCATCATCTCTTATTATTACAGA	53
genomic sequence cloning from At2g22920 from C24 and Col0		
TT1f_v2-FW	TGGACTAGTACGAGAATTGCAAAG	24
TT1f_v2-REV	ACCGGGAAGAAACTTGACGA	20
TT2f_v4-FW	CGTGGAGCGACCAGTGAT	18
TT2f_v4-REV	CTTCACGTACCCTCTCGTCA	20
TT3f_v2-FW	CTTATGCAGGGCTACCACAGT	21
TT3f_v2-REV	ACCACTGTTTACCAAAAACCGC	22
TT4f_v1-FW	ATATCATATGAATAACAGCATC	22
TT4f_v1-REV	GCATAGGGCATCATCATCTC	20
* Primer sets amplifying only C24 insertion		
TT222-FW	TGAGCTCATGATTCGACTGAGTTAATG	27

Supplementary Note 1. Spectrometric data of saiginol A.

kaempferol-3-*O*-[2-*O*-(α-L-rhamnopyranosyl)-6-*O*-(sinapoyl)-β-D-glucopyranoside]-7-*O*-α-L-rhamnopyranoside

Yellow amorphous solid. $[\alpha]_D^{20}$ – 54.1° (*c* 0.09, MeOH). UV (MeOH): λ_{max} (logε): 201.5 (3.47), 223.0 (3.61), 266.5 (3.37), 331.5 (3.47). HRESI-MS: *m/z* 969.2637 ($[M+Na]^+$, calcd. for $C_{44}H_{50}O_{23}Na$, 969.2635). ^1H NMR (CD_3OD) δ: 6.25 (1H, br.s, H-6), 6.41 (1H, br.s, H-8), 7.90 (2H, d, *J* = 8.8 Hz, H-2' and H-6'), 6.85 (2H, d, *J* = 8.8 Hz, H-3' and H-5') (kaempferol; H-6, 8, H-2' to 6'), 5.75 (1H, d, *J* = 7.2 Hz, H-1), 3.63 (1H, m, H-2), 3.58 (1H, m, H-3), 3.32 (1H, m, H-4), 3.51 (1H, m, H-5), 4.26 (1H, m, H-6a), 4.40 (1H, m, H-6b) (glucose; H-1 to H-6a,b), 5.23 (1H, br.s, H-1), 3.99 (1H, m, H-2), 3.75 (1H, m, H-3), 3.32 (1H, m, H-4), 3.97 (1H, m, H-5), 0.90 (1H, m, H-6) (rhamnose 1; H-1 to H-6), 6.66 (2H, s, H-2), 6.06 (1H, d, *J*= 16.0, H-7α), 7.28 (1H, d, *J*= 16.0, H-8β), 3.87 (6H, s, $-OCH_3$) (sinapic acid; H-2, H-7α, H-8β and $-OCH_3$), 5.33 (1H, br.s, H-1), 3.47 (1H, m, H-2), 4.04 (1H, m, H-3), 3.76 (1H, m, H-4), 3.47 (1H, m, H-5), 1.18 (1H, m, H-6) (rhamnose 2; H-1 to H-6). ^{13}C NMR (CD_3OD) δ: 161.7, 134.5, 179.7, 159.7, 99.9, 163.2, 96.4, 157.9, 107.5, 123.2, 132.5, 116.4, 163.0, 116.4, 132.5 (kaempferol; C-2 to C-10 and C-1' to C-6'); 100.2, 80.2, 79.2, 74.3, 76.1, 64.6 (glucose; C-1 to C-6); 102.8, 70.4, 72.5, 72.6, 72.6, 17.2 (rhamnose 1; C-1 to C-6), 12.75, 107.0, 148.5, 139.5, 149.5, 107.0, 115.6, 147.0, 168.7 (sinapic acid; C1- to C-9), 99.9, 71.4, 71.8, 72.3, 73.9, 19.3 (rhamnose 2; C-1 to C-6). HMBC correlations from the anomeric proton (Rha1 H-1, δ 5.23) to C-2 of glucose, the protons (glucose H-6a,b, δ 4.26 and 4.40) to C-9 of sinapic acid and the anomeric proton (Rha2 H-1, δ 5.33) to C-7 of kaempferol were observed.
